# Two-Stage Machine Learning-Based Approach to Predict Points of Departure for Human Noncancer and Developmental/Reproductive Effects

**DOI:** 10.1021/acs.est.4c00172

**Published:** 2024-05-02

**Authors:** Jacob Kvasnicka, Nicolò Aurisano, Kerstin von Borries, En-Hsuan Lu, Peter Fantke, Olivier Jolliet, Fred A. Wright, Weihsueh A. Chiu

**Affiliations:** † Department of Veterinary Physiology and Pharmacology, Interdisciplinary Faculty of Toxicology, Texas A&M University, College Station, Texas 77843, United States; ‡ Quantitative Sustainability Assessment, Department of Environmental and Resource Engineering, Technical University of Denmark, Bygningstorvet 115, 2800 Kgs. Lyngby, Denmark; § Departments of Statistics and Biological Sciences and Bioinformatics Research Center, North Carolina State University, Raleigh, North Carolina 27695, United States

**Keywords:** QSAR model, machine learning, toxicity prediction, chemical risk assessment, high-throughput screening, life cycle impact assessment (LCIA)

## Abstract

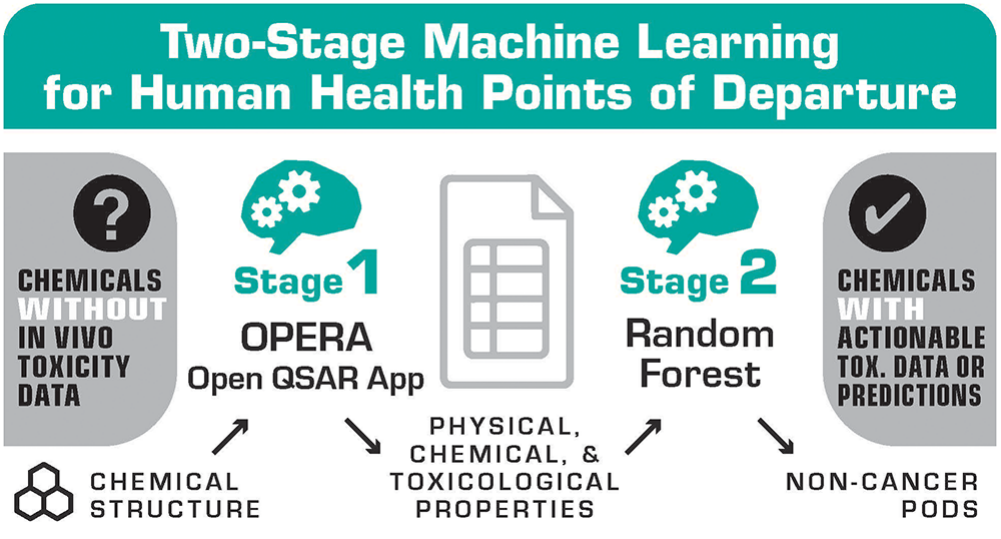

Chemical points of departure (PODs) for critical health effects are crucial for evaluating and managing human health risks and impacts from exposure. However, PODs are unavailable for most chemicals in commerce due to a lack of *in vivo* toxicity data. We therefore developed a two-stage machine learning (ML) framework to predict human-equivalent PODs for oral exposure to organic chemicals based on chemical structure. Utilizing ML-based predictions for structural/physical/chemical/toxicological properties from OPERA 2.9 as features (Stage 1), ML models using random forest regression were trained with human-equivalent PODs derived from *in vivo* data sets for general noncancer effects (*n* = 1,791) and reproductive/developmental effects (*n* = 2,228), with robust cross-validation for feature selection and estimating generalization errors (Stage 2). These two-stage models accurately predicted PODs for both effect categories with cross-validation-based root-mean-squared errors less than an order of magnitude. We then applied one or both models to 34,046 chemicals expected to be in the environment, revealing several thousand chemicals of *moderate* concern and several hundred chemicals of *high* concern for health effects at estimated median population exposure levels. Further application can expand by orders of magnitude the coverage of organic chemicals that can be evaluated for their human health risks and impacts.

## Abstract 2

Most chemicals lack toxicity data related to human health. This study uses machine learning to fill this gap, greatly expanding the ability to characterize chemical risks and impacts.

## Introduction

Determining a chemical’s point of departure (POD) is crucial to evaluating and managing health risks and toxicity impacts associated with chemical exposure. The POD is the starting point along the dose–response curve for extrapolating health risks to relevant exposure levels that may be encountered in the general population.^1^ A variety of impact and risk assessment frameworks, such as contaminated site remediation, life cycle impact assessment (LCIA), chemical alternatives assessment (CAA), and health-based risk screening, heavily rely on PODs.^2,3^ These PODs are primarily developed in regulatory or other authoritative assessments by agencies, such as the United States Environmental Protection Agency (U.S. EPA), that synthesize available toxicity data from *in vivo* studies and identify the “critical” or “most-sensitive” end point for characterizing health effects. However, due to the resource-intensive nature of these assessments, such authoritative PODs are available for less than 1,000 chemicals, which is a tiny fraction of the more than 150,000 commercial chemicals to which humans may be exposed.^4,5^ Consequently, most of these chemicals lack comprehensive human health assessments and are not included in impact and risk assessment tools, such as USEtox.^6^

To partially address the lack of authoritative assessments, a number of open-source databases compiling publicly available experimental *in vivo* toxicity data required for POD derivation have emerged, such as the U.S. EPA’s Toxicity Value Database (ToxValDB)^7^ and the European Chemicals Agency’s International Uniform Chemical Information Database (IUCLID; https://iuclid6.echa.europa.eu/). These databases have enabled researchers to derive “surrogate” PODs, through rigorous curation and statistical approaches, as a proxy for PODs that would be selected in an authoritative assessment.^8^ However, even though use of these databases increases the availability of PODs by an order of magnitude to about ten thousand chemicals, the remaining gap underscores the need for a high-throughput approach to develop surrogate PODs in the absence of *in vivo* data.

“New approach methods” (NAMs), including *in vitro* and computational (*in silico*) approaches, have emerged as promising, high-throughput alternatives to animal testing while also addressing ethical concerns regarding animal use. A prime example of *in silico* NAMs is QSAR (Quantitative Structure–Activity Relationship) modeling. QSAR models commonly use machine learning (ML) to predict biological activity based on chemical structure information. Applications of QSAR modeling have substantially expanded the availability of toxicologically relevant data. For example, Mansouri et al. developed a collection of open-source ML models known as “OPERA” [Open (Quantitative) Structure–activity/property Relationship App].^9,10^ These models predict structural and physical–chemical properties, environmental fate metrics, acute toxicity, and toxicokinetic end points for hundreds of thousands of chemicals. Many of these predictions are available through open-source web platforms such as the CompTox Chemistry Dashboard by U.S. EPA^11^ and the National Toxicology Program (NTP) Integrated Chemical Environment (ICE).^12^

Previous studies have also developed QSAR models to predict PODs. For instance, the models developed by Wignall et al. included those that predict PODs, such as benchmark doses (BMDs) and No Observed Adverse Effect Levels (NOAELs), using training data from several hundred chemicals with available authoritative human health assessments (*n* = 137 for BMDs and *n* = 487 for NOAELs).^4^ For these PODs, the models by Wignall et al.^4^ explained between 28% and 45% of the variance, with mean absolute errors of 0.93–1.13 log_10_-units. Pradeep et al. used a similar approach to predict effect levels for specific species-study type combinations in ToxValDB, with training sets ranging in size from <100 to over 3600 and a wide range of performance depending on the study type.^13^ Combining all study types, they achieved an *R*^2^ of 0.53 and RMSE of 0.71 in log_10_-units, but their approach does not provide surrogate PODs that reflect the “critical” or “most-sensitive” end points for characterizing health effects. Thus, a substantial gap remains in the availability of surrogate PODs for a wider range of chemicals.

Conventional ML-based QSAR models often rely on hundreds of molecular descriptors as features.^4,13^ While these descriptors can enable accurate predictions and many have good structural interpretability, it can be challenging to explain their toxicological importance to practitioners and decision-makers. Recognizing this challenge, the Organisation for Economic Co-operation and Development’s (OECD) *(Q)SAR Assessment Framework*(14) includes a key “mechanistic interpretation” criterion for evaluating a QSAR model, defined as “how the rationale behind a (Q)SAR model is consistent with or accounts for the knowledge related to the predicted property.” This guidance highlights the importance of QSAR models that not only predict accurately but also provide insights into their underlying scientific basis to enhance their utility and trustworthiness. Thus, in accordance with the OECD report suggesting preference for a “physical-chemical interpretation (if possible) that is consistent with a known mechanism of biological action”, we posit that the structural/physical/chemical/toxicological properties that are available in OPERA, such as water solubility and bioconcentration factor, are more easily understood by a typical practitioner than typical chemoinformatic descriptors and offer a path toward more “understandable” machine learning.

Building on prior efforts, this study aimed to expand the coverage of chemicals with toxicity values that can be used as surrogates for human-equivalent noncancer PODs for oral exposure in the absence of *in vivo* data. Our objectives were 3-fold:

1. Develop and evaluate a two-stage QSAR modeling framework that incorporates an intermediate layer of structural/physical/chemical/toxicological properties as features.
2. Generate an extended set of oral surrogate PODs, with quantified model prediction errors based on cross-validation, for a wide range of chemicals.
3. Apply this framework to a large data set of chemicals observed in the environment, assessing potential health risks using the margin of exposure as a metric.

Following Aurisano et al.,^8^ we differentiated between reproductive/developmental and nonreproductive/developmental effects (“general noncancer effects”).^3,15^ The surrogate PODs from this study can be integrated into various chemical management and exposure and impact assessment frameworks for health-based risk screening, LCIA, CAA for chemical substitution, and exposure and risk prioritization.^3,16,17^

## Methods

**Figure 1:**
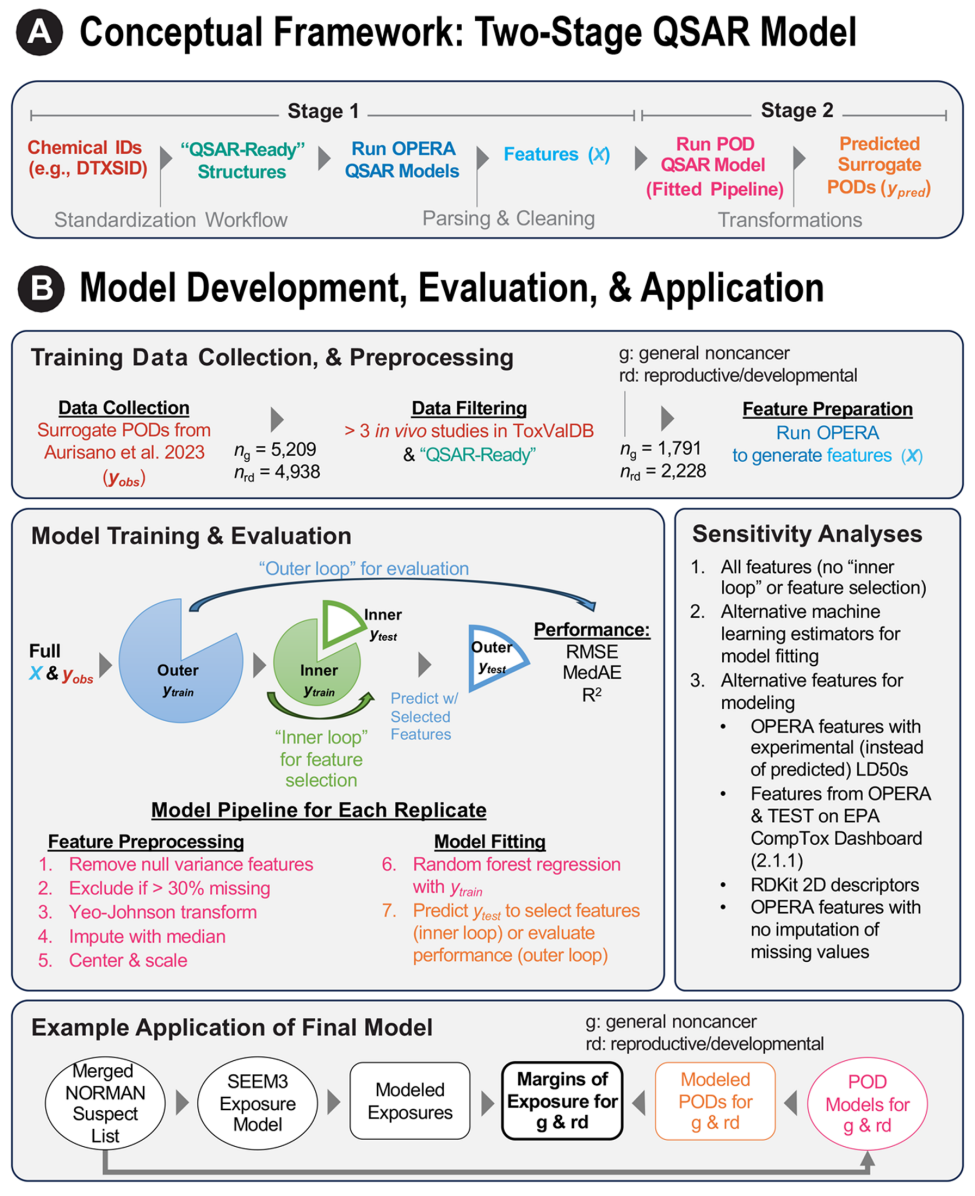
Overview of the two-stage machine learning framework for predicting points of departure. (A) Conceptual framework. (B) Model development, evaluation, and application. The surrogate points of departure were obtained from Table S5 of Aurisano et al.^8^ Features were extracted from predictions by OPERA 2.9.^9,10^Figures S1–S2 provide an overview of the model training and evaluation. Exposure estimates were obtained from SEEM3 by Ring et al.^19^ Application chemicals were expected to occur in the environment and lacked *in vivo* points of departure.^20,21^ Note: ML, machine learning; POD, point of departure; QSAR, quantitative structure–activity relationship; OPERA, OPEn structure–activity/property Relationship App; ToxValDB, Toxicity Value Database; RMSE, root-mean-squared error; MedAE, median absolute error; *R*^2^, coefficient of determination; MAD, median absolute deviation; SEEM, Systematic Empirical Evaluation of Models.

To address the stated objectives, we developed a two-stage ML framework. The first stage derives ML-based predictions for structural, physical, chemical, and toxicological properties that are readily interpretable. The second stage leverages these properties as features in a separate ML model to predict surrogate PODs. Figure 1A illustrates the conceptual framework, while Figure 1B shows an overview of the model development, evaluation, and application. The conceptual framework comprises the following steps:

1. Select and identify chemicals for modeling.
2. Standardize chemical structures to make them “QSAR-ready”.
3. Run prior QSAR models for feature extraction (Stage 1).
4. Clean and parse the QSAR predictions to obtain raw features.
5. Apply these features in a modeling pipeline to predict PODs (Stage 2).

All ML algorithms for predicting PODs were implemented using Python 3.9, leveraging open-source libraries such as scikit-learn 1.2.2.^18^ The source code, results, and input files associated with this study are openly available in a GitHub repository at https://github.com/jkvasnicka/Two-Stage-ML-Oral-PODs.

### Training Data Collection and Preprocessing

#### Data Collection

Predicting PODs was essentially a regression task with a continuous target vector  of oral doses, in log10-transformed units of mg·(kg-d)^−1^, representing a POD for a given effect category *e*, and inputs represented by a matrix **X**, where each row corresponds to a sample and each column corresponds to one of *n* distinct features, i.e., . This task required labeled data involving mapping of chemical identifiers to their respective *in vivo* PODs. Specifically, we used the surrogate oral PODs from Table S5 of Aurisano et al.,^8^ which were derived through meticulous curation and statistical analysis of *in vivo* experimental animal data from ToxValDB 9.1,^7^ adjusted to chronic human equivalent benchmark doses (BMDh). Throughout this study, the U.S. EPA’s DSSTox Substance Identifier (DTXSID) uniquely identifies each chemical.

#### Data Filtering

Initially, there were 5,209 unique chemicals with surrogate PODs for general noncancer effects and 4,938 chemicals for reproductive/developmental effects. However, a series of filtering steps removed chemicals that were unsuitable for modeling (Figure 1B). First, chemicals with ≤3 *in vivo* studies were excluded because those surrogate PODs may be less robust (Aurisano et al.^8^ used the lower 25th percentile of the distribution of available PODs for a chemical as the surrogate POD), leaving 2,404 and 2,999 chemicals for the respective effect categories. Next, a general applicability domain exclusion and standardization workflow was applied to generate “QSAR-ready” structures compatible with a variety of modeling approaches.^22,23^ Applying this workflow yielded 1,791 organic chemicals for general noncancer effects and 2,228 organic chemicals for reproductive/developmental effects.

#### Feature Extraction and Preparation

To obtain features, we leveraged the QSAR modeling framework, OPERA 2.9, by Mansouri et al.^9,10^ Specifically, we used the command-line version, OPERA2.9_CL, and input the chemical identifiers (DTXSID) as a text file. OPERA then retrieved the corresponding QSAR-ready structures as simplified molecular-input line-entry system (SMILES) strings from its internal database. This execution yielded 39 interpretable features (e.g., water solubility) with feature-specific applicability domain information. We then flagged features outside the applicability domain as “missing” if both of the following criteria by Mansouri et al.^9^ were met:

1. The value was outside the *global* applicability domain of the model/feature.
2. The value had a low *local* applicability domain index (<0.4) with respect to its nearest neighboring values.

Figure S3 displays the distributions of raw features for all chemicals in this study, with corresponding descriptions in Table S3. Given the diverse nature of these features, we designed a robust feature preprocessing pipeline for feature transformation (Figure 1B), which can be generalized across a variety of ML estimators, as detailed below.

### Model Training and Evaluation

#### Overview of Modeling Pipeline

The QSAR models for predicting PODs consisted of a pipeline of feature preprocessing steps and an ML estimator (e.g., random forest) (Figure 1B). This design ensured that transformation parameters (e.g., median for imputation) were derived solely from the training data, minimizing potential for data leakage and overoptimistic performance estimates. The feature preprocessing steps are described in the Supporting Information (see section, *Feature Preprocessing Steps*) and include imputation of missing values using the median (features were excluded if >30% imputation would be necessary). For the last components in the pipeline (steps 6 and 7 in Figure 1B), we chose the Random Forest Regressor and made predictions for the surrogate PODs. This estimator was a reasonable choice, given its track record of robust performance without extensive preprocessing or hyperparameter tuning^24^ and its successful applications in prior studies involving POD prediction.^4,13^ The algorithm constructs a collection of decorrelated decision trees using bootstrapped sampled versions of the training data and then averages predictions to minimize variance.^25^ For the hyperparameters, we used the scikit-learn 1.2.2 defaults,^18^ except for the number of features to consider when searching for the best split, which we set to 1/3 (or at least 1) of the available features,^24^ instead of considering all features.

For model training and evaluation, we implemented nested 5-fold cross-validation, with separate “inner” and “outer” loops (Figures 1B, S1, and S2). The “inner” loop is used for feature selection, whereas the “outer” loop is used to evaluate performance. Thus, for an iteration of the “outer” loop, the data are divided into an “outer” training and testing data set. The “outer” training set is sent to the “inner” loop where it is repeatedly divided into “inner” training and testing data sets. This “inner” loop trains an “inner” model in order to conduct feature selection (described below under Model Training with Feature Selection). The selected features are then passed back to the “outer” loop, which trains a model using only those selected features with the “outer” training data set and evaluates performance using the “outer” testing data. This whole process is then repeated multiple times with different randomizations (described below under Model Evaluation).

#### Model Training with Feature Selection

Given the 39 features from OPERA 2.9 (Figure S3),^9,10^ we hypothesized that a subset of 10 features would be sufficient for successful modeling while remaining interpretable. We selected the value of “10” *a priori* to avoid overfitting and verified this hypothesis in a sensitivity analysis (described below) where all features were used without feature selection. If the value of “10” were to materially degrade performance, then we could have used more complex feature selection approaches, such as recursive feature elimination.

To select features in an objective, robust, and reproducible manner, we implemented a feature selection scheme by nesting a permutation feature importance algorithm within a repeated k-fold cross-validation loop. Specifically, we repeatedly divided the data into 5-folds, training the model on 4/5 of the data in which the algorithm measured feature importance by assessing the decrease in model performance upon random permutation of feature values. In particular, we used the median value for this importance score across random permutations as the selection criterion. The cross-validation loop minimized biases and overoptimistic performance scores. Further details can be found in the Supporting Information (see section *Model Training Steps* and Figure S1).

#### Model Evaluation

To gauge the model’s generalization to unseen data, we nested the training process described above within another repeated *K*-fold cross validation loop. For this loop, we used 30 repetitions and 5-folds, yielding 150 (30 × 5) replicate models that underwent the same model training steps. To quantify performance, we used the root-mean-squared error (RMSE), median absolute error (MedAE), and coefficient of determination (*R*^2^). Further details regarding the model evaluation, along with definitions of the performance metrics, can be found in the Supporting Information (see section *Model Performance Metrics* and Figure S2).

#### Model Benchmarking

To further evaluate our models, we benchmarked the QSAR-derived PODs (POD_QSAR_) against estimates from other studies. Specifically, we referenced the original authoritative PODs (POD_authoritative_) and the target variable of surrogate PODs (POD_surrogate_) from Aurisano et al.,^8^ both of which were fully adjusted to BMDh. Additionally, we compared our POD_QSAR_ values with oral equivalent doses derived from combining high-throughput *in vitro* bioactivity data with toxicokinetic data by using reverse dosimetry. Specifically, we used the “POD_NAM,50_” values from Table S2 of Paul Friedman et al.,^26^ where “50” denotes the median from a population distribution of steady-state administered equivalent doses. POD_NAM,50_ values were available for 263 chemicals for general noncancer effects and 13 chemicals for reproductive/developmental effects.

### Sensitivity Analysis

We conducted a sensitivity analysis to assess generalization error sensitivity to different data sets, feature preprocessing, and ML estimators. Our baseline Final Model was described above, involving feature selection among all 39 OPERA 2.9 features, imputation of missing values, and the Random Forest Regressor. We compared several additional models for each effect category using the same evaluation scheme described above (Figure S2), varying one modeling aspect at a time. These alternative models are shown in Figure 1 (see *Sensitivity Analyses*), and corresponding descriptions are in Table S1. All models were applied to the same chemicals, except the model involving no imputation, which was restricted to those chemicals with no missing feature values (*n* = 184–227).

### Model Application

We demonstrated application of our final two-stage models using a large data set of organic chemicals expected to occur in the environment and for which human oral exposure could be estimated. Specifically, we assessed 34,809 chemicals that were on the Merged NORMAN Suspect List (SusDat)^20,21^ and within the applicability domain of SEEM3 (Systematic Empirical Evaluation of Models) by U.S. EPA.^19^ We excluded any chemicals outside the “general applicability domain” due to their being unsuitable for QSAR modeling based on the standardization workflow mentioned above^22,23^ and that had a POD_surrogate_ value used for model training (“training chemicals”). This exclusion resulted in 33,407 chemicals predicted for general noncancer effects and 32,970 chemicals predicted for reproductive/developmental effects (34,046 chemicals across the two sets of predictions). We also evaluated how these chemicals fit within the “feature-specific applicability domains” of the OPERA models and the extent to which the distribution of features compared to that of the training set chemicals.

The margin of exposure was used as a health risk metric to compare SEEM3 predicted population median oral exposures [*ŷ*_exposure,*i*_ in mg·(kg-d)^−1^] with the QSAR-predicted POD [POD_QSAR,*i*_, also in mg·(kg-d)^−1^]. For each sample *i*, the margin of exposure (MOE_*i*_) was calculated as
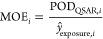
1

We screened chemicals for potential health concerns using the following categorization scheme:^27,28^

1. Low concern for the median population exposure: MOE_*i*_ > 100
2. Moderate concern for the median population exposure: 1 < MOE_*i*_ ≤ 100
3. High concern for the median population exposure: 0 < MOE_*i*_ ≤ 1

SEEM3 exposure predictions (*ŷ*_exposure,*i*_) for an individual at the population median exposure, accompanied by a model-based Bayesian 90% credible interval representing uncertainty,^19^ were downloaded from ICE.^12^ We also assessed the contribution of POD_QSAR_ (hazard) uncertainty to the overall uncertainty in the margin of exposure in addition to exposure uncertainty from SEEM3. Specifically, we derived 90% prediction intervals of the POD_QSAR_ uncertainty for each percentile of exposure uncertainty for the median individual. The derivation of these prediction intervals is shown in the Supporting Information (see *Margin of Exposure Uncertainty Analysis*).

## Results

### Data Set Characterization

The proportions of missing values across all 39 features from OPERA 2.9 for the training chemicals and for the application chemicals can be found in the Supporting Information (Figure S4). Most features predominantly had samples within their respective applicability domains. However, three features had more than 30% missing values and were subsequently removed in the pipeline.

### Performance Evaluation and Benchmarking

**Figure 2:**
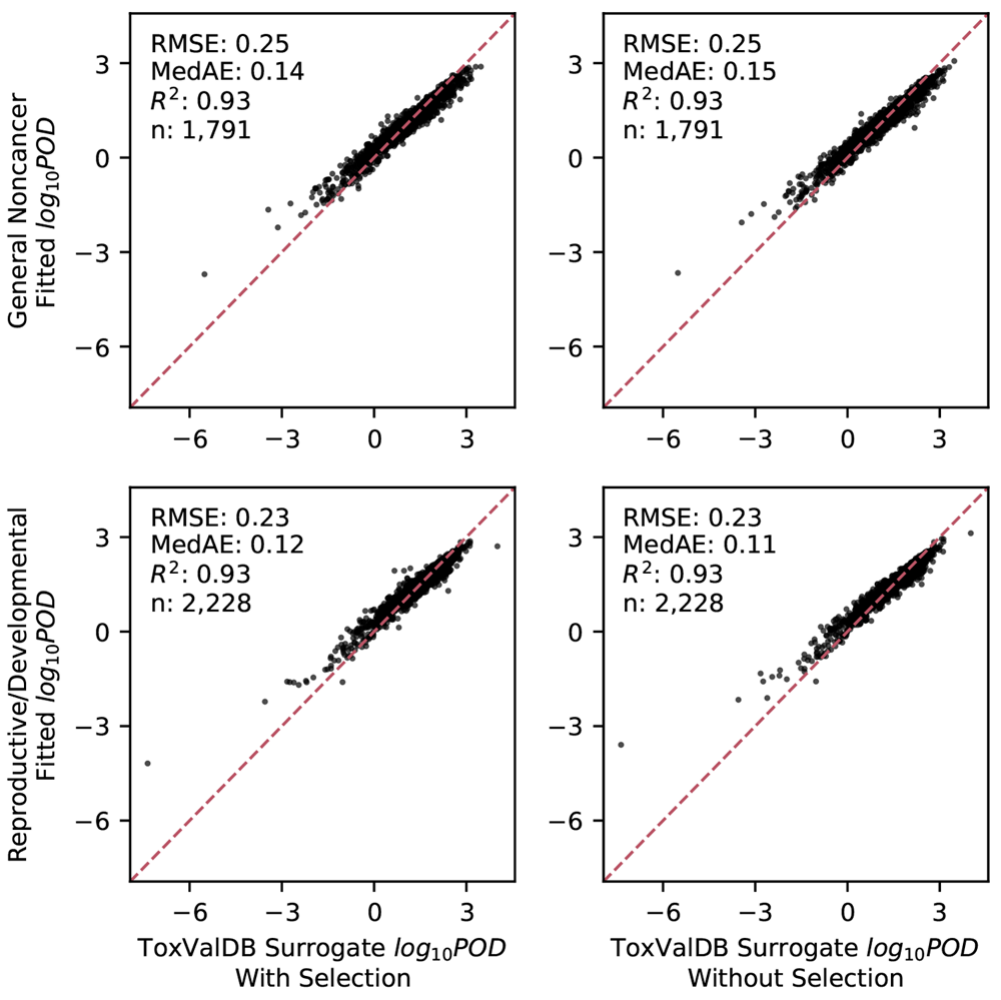
Model fitting. In-sample performance is assessed through scatterplots and performance metrics comparing the fitted and observed values for each chemical The fitted values are predictions from the cross-validated final models that were fitted on the full labeled data set. The figure is subdivided by target effect category and by whether the feature selection was implemented. Note: RMSE, root-mean-squared error; MedAE, median absolute error; *R*^2^, coefficient of determination; *n*, sample size.

**Figure 3:**
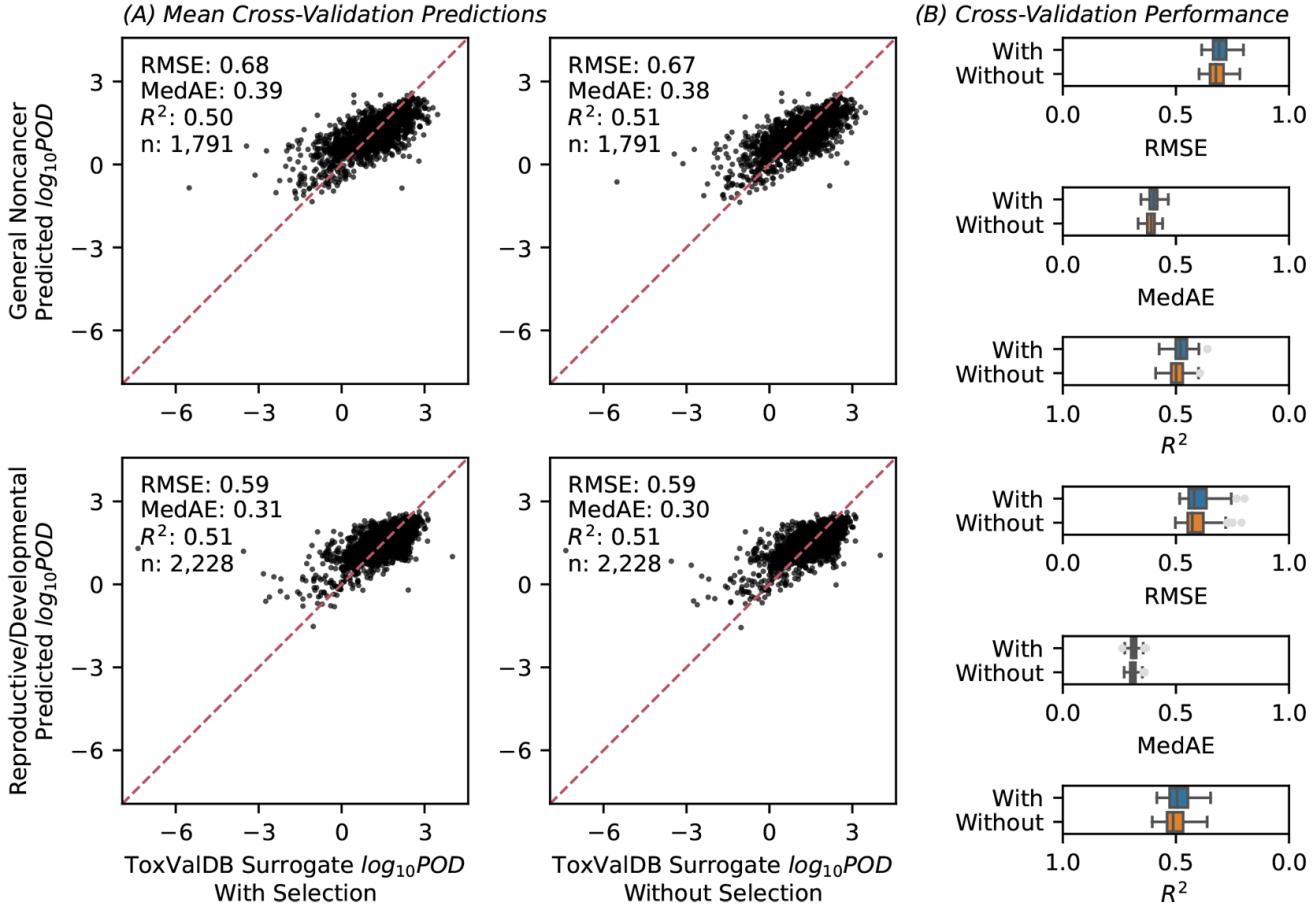
Model evaluation. (A) Out-of-sample performance is assessed through scatterplots comparing the mean predicted values for each chemical when it is part of the “testing” data set across 30 cross-validation repeats (*y*-axis) against the corresponding surrogate values (*x*-axis). The dashed red line indicates perfect correspondence. (B) The distribution of performance metrics from 150 cross-validation scores (30 repeats × 5-fold), where each boxplot shows the median and interquartile range with whiskers representing the 95% confidence interval. The figure is subdivided by the performance metric, target effect category, and by whether feature selection was implemented. Note: RMSE, root-mean-squared error; MedAE, median absolute error; *R*^2^, coefficient of determination; *n*, sample size. The scale for *R*^2^ is reversed to be consistent with values to the “left” corresponding to better performance.

The final models accurately fitted/predicted POD_surrogate_ values for both effect categories, as shown by their RMSE, MedAE, and *R*^2^. The models demonstrated consistent performance for both effect categories regardless of feature selection. Because of our nested cross-validation approach, each chemical may be part of the “training” or the “testing” data set depending on the replicate. Figure 2 summarizes the “in-sample” model fitting, showing the predictions of the cross-validated final models that were fitted on the full labeled data set. The accuracy was demonstrated by the clustering of fitted predictions and observations along the diagonal line, the low values for the dispersion measures (RMSE, MedAD), and the high *R*^2^ values. More importantly, Figure 3 summarizes the “out-of-sample” results, where the median prediction shown is across replicates when the chemical is part of the “testing” data set. The estimated generalization errors (with 5th to 95th percentiles) based on cross validation were also quite good. These results imply that, for a “new” chemical, we can expect the model to predict the POD with a GSD error of less than 3.5- to 5.7-fold (taking the range of RMSE values from 0.54 to 0.76) or equivalently a 95% confidence interval spanning 11- to 30-fold in each direction.

The benchmarking revealed that the POD_QSAR_ values correlated well with the corresponding POD_authoritative_ values for general noncancer effects (*n* = 564) (Figure S5), with RMSE = 0.50 and MedAE = 0.32, both in log10-units, and *R*^2^ = 0.79. The correspondence was poorer for reproductive/developmental effects, with RMSE = 0.75, MedAE = 0.40, and *R*^2^ = 0.47. For both effect categories, the POD_QSAR_ values corresponded substantially better to the POD_authoritative_ values than did the POD_NAM,50_ values that were derived from *in vitro* bioactivity data.^26^ The POD_NAM,50_ values yielded negative *R*^2^ values, indicating worse performance than that of a naïve constant model. However, the performance of POD_QSAR_ values in this comparison may be overstated because they incorporated information about POD_authoritative_ indirectly through the use of surrogate PODs derived from ToxValDB, while the POD_NAM,50_ consisted of a completely independent data set.

### Feature Importance

Results from the feature selection can be found in the Supporting Information (Figures S6–S10). Notably, the most important feature was consistently the QSAR-predicted LD50 derived from *in vivo* rat acute oral toxicity studies.^29^ Four important features were common to both effect categories:

- QSAR-predicted LD50 derived from *in vivo* rat acute oral toxicity studies (CATMoS_LD50_pred)
- Combined dipolarity/polarizability (CombDipolPolariz)
- Ready biodegradability, a binary variable (ReadyBiodeg_pred_discrete)
- Water solubility at 25 °C (WS_pred)

For these features, no more than 11% of the training data sets were imputed, with less than 1% imputed for the predicted LD50 (Figure S4). The remaining important features depended on the effect category (Figures S6–S10) and involved the imputation of no more than 25% of the training set. Some additional important features were identified by the replicate models but were excluded from the final models to prevent overfitting (Figure S6).

### Sensitivity Analysis

Table 1 compares the estimated generalization errors of the models from the sensitivity analysis. The best overall performance was exhibited by the baseline model (all 39 OPERA 2.9 features, imputation of missing values, Random Forest Regressor). However, as mentioned, this model’s performance was indistinguishable from the final model that involved a subset of 10 important features (Figure 3B). Interestingly, when the baseline model was applied to samples without the need for imputation, the model continued to exhibit favorable performance in terms of RMSE and MedAE but with substantially higher variances and with *R*^2^ values that were much lower (Table 1), likely due to the much more limited training sample sizes. Additionally, when using the more “traditional” descriptors from RDKit (2022.09.5),^30^ the performance was similar to, but slightly poorer than, our baseline model, suggesting that the 10 selected OPERA features encapsulate the essential information for POD prediction. Overall, our final model (Random Forest Regressor with feature selection and OPERA 2.9 features) was among the highest performing models in terms of its combination of a low prediction error (RMSE and MedAE) and higher *R*^2^.

**Table 1:** Comparison of Performance Metrics for QSAR Models Predicting Points of Departure^a^

QSAR model (*n*)	RMSE	MedAE	*R*^2^
Current Work: General Noncancer Effects			
**RandomForestRegressor with feature selection (1,791)**	0.69 [0.64–0.76]	**0.40 [0.37–0.44]**	**0.48 [0.41–0.53]**
bRandomForestRegressor (1,791)	0.68 [0.62–0.74]	0.39 [0.35–0.43]	0.50 [0.44–0.56]
bGradientBoostingRegressor (1,791)	0.69 [0.64–0.75]	0.41 [0.37–0.46]	0.48 [0.42–0.55]
bRidge (1,791)	0.73 [0.68–0.79]	0.44 [0.40–0.48]	0.42 [0.36–0.48]
bLinearRegression (1,791)	0.73 [0.68–0.79]	0.44 [0.40–0.48]	0.42 [0.36–0.48]
bXGBRegressor (1,791)	0.72 [0.66–0.78]	0.42 [0.38–0.46]	0.43 [0.36–0.51]
bSVR (1,791)	0.96 [0.89–1.04]	0.64 [0.57–0.69]	–0.01 [−0.03 to 0.01]
bMLPRegressor (1,791)	2.75 [1.56–5.53]	0.67 [0.58–0.84]	–7.50 [−36.72 to −1.72]
cOPERA w/Exp. LD50s (1,791)	0.69 [0.63–0.75]	0.40 [0.37–0.43]	0.48 [0.42–0.55]
cCompTox Features (1,791)	0.75 [0.69–0.82]	0.44 [0.39–0.49]	0.39 [0.31–0.46]
cRDKit Features (1,789)	0.71 [0.65–0.78]	0.40 [0.36–0.44]	0.45 [0.38–0.51]
cNo Imputation (184)	0.58 [0.46–1.17]	0.37 [0.28–0.49]	0.22 [0.02–0.44]
Current Work: Reproductive/Developmental Effects			
**RandomForestRegressor with feature selection (2,228)**	**0.58 [0.54–0.72]**	**0.31 [0.28–0.34]**	**0.49 [0.38–0.56]**
bRandomForestRegressor (2,228)	0.57 [0.53–0.72]	0.31 [0.29–0.35]	0.51 [0.40–0.58]
bGradientBoostingRegressor (2,228)	0.59 [0.54–0.73]	0.32 [0.30–0.35]	0.49 [0.37–0.55]
bRidge (2,228)	0.63 [0.58–0.76]	0.37 [0.34–0.40]	0.42 [0.32–0.48]
bLinearRegression (2,228)	0.63 [0.58–0.76]	0.37 [0.34–0.40]	0.42 [0.32–0.48]
bXGBRegressor (2,228)	0.62 [0.56–0.74]	0.33 [0.30–0.36]	0.43 [0.34–0.52]
bSVR (2,228)	0.85 [0.77–0.96]	0.54 [0.51–0.58]	–0.03 [−0.06 to −0.01]
bMLPRegressor (2,228)	1.75 [1.18–2.71]	0.56 [0.48–0.68]	–3.43 [−10.68 to −0.92]
cOPERA w/Exp. LD50s (2,228)	0.57 [0.53–0.71]	0.32 [0.29–0.34]	0.52 [0.42–0.58]
cCompTox Features (2,228)	0.67 [0.60–0.81]	0.38 [0.34–0.41]	0.34 [0.26–0.44]
cRDKit Features (2,224)	0.62 [0.55–0.73]	0.32 [0.29–0.35]	0.45 [0.37–0.52]
cNo Imputation (227)	0.45 [0.35–0.55]	0.28 [0.20–0.35]	0.40 [0.21–0.53]
Previous Work			
Wignall et al.^4^ NOAEL (487)	N.R.	0.70 [0.06–1.82]	0.45
Pradeep et al.^13^ CHR R,M (11201)	0.92–0.94	N.R.	0.39–0.40
Pradeep et al.^13^ REP R,M (5951)	0.79–0.91	N.R.	0.26–0.31
Pradeep et al.^13^ DEV R,M, Rb (9945)	0.76–0.80	N.R.	0.26–0.29
Pradeep et al.^13^ ALL (71,020)	0.67–0.70	N.R.	0.54–0.57

a **Bold** represents the “final” model used for predictions. Abbreviations: RMSE, root-mean-squared error; MedAE, median absolute error; *R*^2^, coefficient of determination; N.R., not reported; CHR, chronic; REP, reproductive; DEV, developmental; R, rat; M, mouse; Rb, Rabbit. Values for current work are median and 90% CI based on “outer” cross-validation replicates (see Methods). Range for Pradeep et al.^13^ based on internal cross-validation and external test set.

b Sensitivity analyses using different machine learning algorithms.

c Sensitivity analyses using different descriptor sets (all using Random Forest Regressor without feature selection).

### Model Application

**Figure 4:**
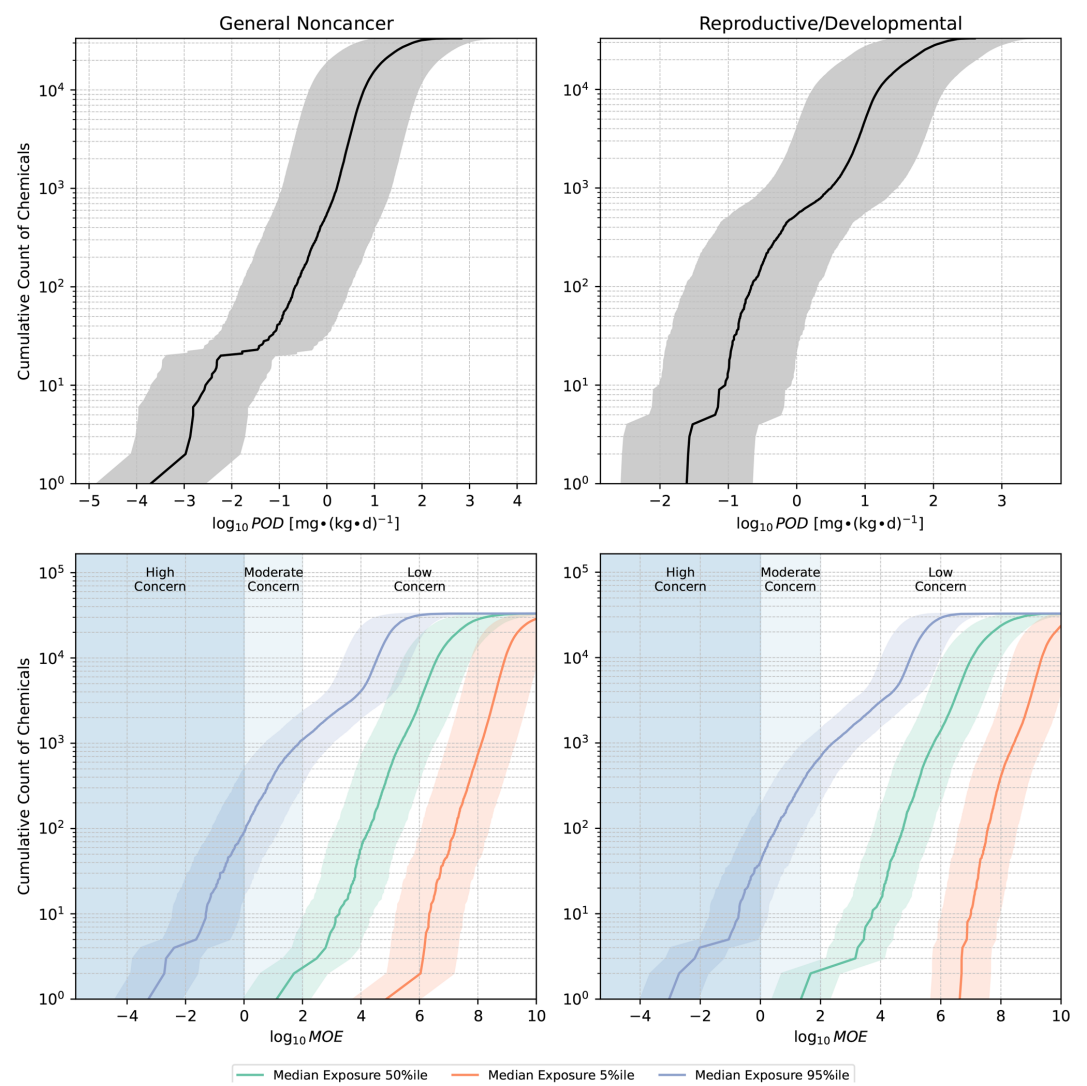
Cumulative counts of the application chemicals in relation to the predicted points of departure and margins of exposure. Data are shown for chemicals that were on the Merged NORMAN Suspect List (SusDat)^20,21^ and within the applicability domain of SEEM3 (*n* = 32,524),^19^ excluding any training chemicals. The margins of exposure correspond to an individual at the population median exposure. Uncertainty is represented in two ways: (1) Exposure uncertainty, reflected by examining margins of exposure at different exposure percentiles; (2) Point of departure (hazard) uncertainty, represented by a 90% prediction interval derived from the median RMSE based on cross validation. Vertical spans highlight different risk categories, as described in the Methods. The *x*-axis is truncated at log_10_MOE = 10. Note: POD, point of departure; MOE, margin of exposure.

The top panels of Figure 4 display cumulative counts of the application chemicals in relation to the corresponding POD_QSAR_ values, along with uncertainty estimates in the form of a 90% prediction interval representing POD_QSAR_ (hazard) uncertainty (Supporting Information eq S8). For general noncancer effects, the median POD_QSAR_ (with 5th to 95th percentiles) was 11 mg·(kg-d)^−1^ (0.82–150). This distribution is somewhat higher (less potent) than that of the available regulatory/authoritative PODs (see Figure S11), as it is expected that higher potency (lower POD) chemicals would be more likely to have such regulatory or authoritative assessments. Additionally, as a sensitivity analysis, we also applied the model without feature selection to these chemicals and obtained consistent results [high correspondence between with and without feature selection: *R*^2^ ∼ 0.9 and RMSE < 0.2 log-10 units (Figure S12)].

The lower panels of Figure 4 show the margins of exposure for an individual at the population median exposure, incorporating the 90% confidence interval for the population median exposure from SEEM3.^19^ About ∼2,400 chemicals emerged as *moderate* concerns for population median exposures (MOE < 100) for general noncancer effects based on the upper 95th percentile of exposure uncertainty estimates and the lower boundary of the 90% prediction interval of POD_QSAR_ uncertainty. In a similar manner, ∼500 chemicals emerged as *high* concerns (MOE < 1) for general noncancer effects. For reproductive/developmental effects, the median POD_QSAR_ was 31 mg·(kg-d)^−1^ (3.4–280), with ∼1,500 chemicals emerging as *moderate* concerns and ∼190 chemicals emerging as *high* concerns. In both cases, most chemicals appear to have low concern MOE values of >100 at the level of the median population exposures. It is however important to note that this level of concern could be substantially higher for subpopulations that regularly use products containing the considered chemicals.^31^ A graphical user interface will be made available for accessing these predictions and identifying chemicals of concern.

Exposure uncertainty was the primary driver of overall uncertainty in the margin of exposure (Figure 4). The typical exposure uncertainty spanned 4 orders of magnitude, evidenced by the median difference in log_10_-transformed exposure estimates between the 95th and 5th percentiles. In contrast, when focusing on POD_QSAR_, the typical error was constrained to less than a factor of 5 according to the median RMSE of ≤0.69 in log10-units (Figure 3B). This error corresponds to a squared geometric standard deviation (GSD^2^) ≤ 23, which, as expected, is larger than the error reported by Aurisano et al.^8^ (GSD^2^ ≤ 17 for all chemicals, GSD^2^ ≤ 14 for chemicals with at least 4 data points) that was based directly on *in vivo* PODs.

## Discussion

This study successfully extended the work of Aurisano et al.,^8^ yielding a two-stage ML framework capable of generating human-equivalent noncancer PODs for oral exposure in the absence of *in vivo* data. This framework was applied to derive surrogate PODs and corresponding margins of exposure for over 30,000 chemicals expected to occur in the environment based on monitoring and which lacked *in vivo* toxicity data.^20,21^ This represents a greater than 3-fold increase in the coverage of organic chemicals with surrogate PODs compared to previous work.^8^ Moreover, a graphical user interface will be made available for accessing predictions for organic chemicals available on the U.S. EPA’s CompTox Chemistry Dashboard that pass the QSAR standardization workflow,^22,23^ which will further increase the coverage of chemicals by over an order of magnitude to ∼800,000.^11^ Moreover, as shown in Figure S4, the rates of imputation for the >30,000 application chemicals were similar to the training set, with the most influential feature (CATMoS_LD50_pred) being imputed for only ∼1% of values. Additionally, our training set of several thousand chemicals from ToxValDB appears to be diverse and representative based on similar coverage of features compared to application chemicals (Figure S13).^7^

Applying our two-stage models revealed several thousand chemicals of *moderate* concern and several hundred chemicals of *high* concern for health effects at estimated median population exposure levels (Figure 4). Notably, the exposure uncertainty was the primary driver of the overall uncertainty in the margin of exposure. Exposure uncertainty was larger than POD_QSAR_ (hazard) uncertainty, despite our QSAR-based approach inherently introducing a larger uncertainty than the surrogate PODs from Aurisano et al. that were based directly on *in vivo* data.^8^ Moreover, we assessed risk only at estimated *median* exposure levels, and for most chemicals, only a small fraction of the population is likely exposed. Thus, the actual uncertainty in exposure is even greater when recognizing the need to address highly exposed subpopulations. These findings underscore the need for refined exposure estimates to better characterize chemical use patterns, product compositions, and human behaviors that influence exposure.^32−34^

In Table 2, we illustrate another case study example, demonstrating how these models could be used in the context of deriving a reference dose (RfD) for a “new” chemical. In particular, we use the example of 4-methylcyclohexanemethanol (MCHM), a chemical used in the processing of coal that spilled from a storage tank into the Elk River in West Virginia, US, in January 2014. At the time, there were no regulatory toxicity values for MCHM. After several days, CDC (2014) developed guidance levels based on a 4-week rat study (Eastman, 1990), and several months later, an expert panel (TERA 2014) proposed refined analyses using the same study.^35−37^ Over six years later, NTP completed a developmental and reproductive toxicity study in rats (NTP 2020).^38^ However, as illustrated in Table 2, utilizing our QSAR models for predicting PODs and deriving RfDs for MCHM would yield very similar results in a much more rapid time frame of minutes, rather than days, months, or years. Additionally, because our predictions include confidence bounds for model uncertainty, they can also be incorporated into probabilistic derivations of toxicity values or health impacts.^39−41^

**Table 2:** Illustration of Application to Deriving a Reference Dose (RfD) for 4-Methylcyclohexanemethanol (MCHM) in the Context of the 2014 Chemical Spill in West Virginia, US

source	point of departure (mg·(kg-d)^−1^)	UF_A_^a^	UF_H_^a^	UF_D_^a^	RfD (mg·(kg-d)^−1^)	analysis time
CDC (2014)^35^	100 (Eastman 1990)	10	10	10	0.1	Days
TERA (2014)^37^	71 (Eastman 1990)^b^	10	10	10	0.07	Months
NTP (2020)^38^	50 (maternal)	10	10	10	0.05	Years
This work: General noncancer	1.9^c^	3^d^	10	1^e^	0.06	Minutes
This work: Reproductive/Developmental	3.5^c^	3^d^	10	1^e^	0.1	Minutes

a Default factor unless otherwise noted. UF_A_ = animal to human; UF_H_ = human variability; UF_D_ = database inadequacy.

b Duration adjusted for 5 days/week exposure.

c QSAR human equivalent POD prediction is 26 [90% CI: 1.9–360] mg·(kg-d)^−1^ for general noncancer and 32 [90% CI: 3.5–290] for reproductive/developmental effects. Lower 95% confidence bound used as a “conservative” POD.

d QSAR predictive POD is already adjusted from animal to human equivalent dose using allometric scaling.

e Reduced to 1 because database uncertainty is already addressed by using lower confidence bound of QSAR-predicted POD and separate predictions for general noncancer and reproductive/developmental effects.

A primary strength of our framework lies in its two-stage approach described in the Methods. Our final models accurately predicted PODs using a subset of 10 interpretable features from OPERA 2.9 (Figure S6).^9,10^ A unique aspect of this approach is the incorporation of predicted biological features. Notably, the QSAR-predicted LD50, derived from *in vivo* rat acute oral toxicity studies,^29^ consistently emerged as the most important feature in our models. For this feature, >99% of the chemicals in the training set was within the applicability domain (Figure S4). This feature indicates the acute mammalian potency of a chemical and was previously predicted with an RMSE of around 0.50 (in log-10 units).^29^ As expected, our POD predictions had RMSE values that were (slightly) greater because they relied on the QSAR-predicted LD50 as a “feature”. Importantly, using *experimental* LD50 values as features in our sensitivity analysis did not materially improve model performance while substantially reducing the applicability domain of the model because only chemicals with experimental LD50s were predicted. Other important features were easily interpretable physical/chemical/biological properties, such as water solubility or fish bioconcentration factor. Moreover, certain structural properties, such as combined dipolarity/polarizability, also emerged as important features independently of the predicted physical/chemical/biological properties. In essence, our two-stage framework is akin to a traditional deep learning model, but providing a supervised intermediate layer that transforms raw chemical descriptors into readily interpretable physical/chemical/toxicological properties. However, a limitation of this approach is that the applicability domain of the overall model is constrained by those of the individual first stage models.

Comparatively, our final models outperformed many alternative models in our sensitivity analyses as well as those published previously. Specifically, our in-sample predictions aligned more closely with authoritative PODs than the combination of high-throughput *in vitro* bioactivity data with toxicokinetic data (Figure S5).^26^ Moreover, even our accuracy for “out-of-sample” predictions was higher than those based on extrapolation from *in vitro*-based PODs. Additionally, as shown in Table 1, our QSAR models had similar or better performance compared to previous models developed by Wignall et al. or Pradeep et al.^4,13^ Although the final “ALL” model by Pradeep et al.^13^ that uses study type and species as additional descriptors had an *R*^2^ value slightly higher than ours, this model includes subchronic and subacute studies and also does not identify a “critical effect” POD. On the other hand, our “surrogate” PODs can be directly used in deriving toxicity values for application in various risk and impact assessment and characterization approaches. Nonetheless, despite differences in target variables making direct comparisons challenging, these studies suggest an upper limit in the performance of QSAR models trained with *in vivo* data from ToxValDB.^7^ Moreover, the performance achievable through QSAR modeling is constrained by the intrinsic variability in the derived toxicity values and PODs across different organizations for identical chemicals.^4^

For regulatory use, it is also important to consider our model and framework in light of internationally recognized evaluation criteria for QSAR models. According to the *(Q)SAR Assessment Framework* by OECD,^14^ a QSAR model under consideration should be associated with (1) a defined end point; (2) an unambiguous algorithm; (3) a defined domain of applicability; (4) appropriate measures of goodness-of-fit, robustness, and predictivity; (5) a mechanistic interpretation, if possible. Table S2 shows the results of applying the *(Q)SAR Assessment Framework* to our modeling framework, demonstrating how our framework conforms to general principles and criteria for use of QSAR models.^14^

Despite its advantages, our framework has several notable limitations. First, it is possible that the actual generalization errors of our models were larger than those reported (Figure 3B), particularly for features with a large proportion of missing values. In our framework, missing values were imputed with the median, a common practice to maintain data set integrity. However, this approach can bias predictions toward central estimates, effectively narrowing the observed variability. This “mean reversion” phenomenon can result in predictions that are less varied and more centered around the median (Figure S14), which might not always reflect the underlying distribution. This problem was partially mitigated by excluding features with many missing values from our modeling pipeline (Figure 1B). Furthermore, based on our in-sample performance and benchmarking, there may be a small trend toward overpredicting PODs for higher potency chemicals (Figures 2 and S5). Again, this may be a mean reversion phenomenon because of random forest is an ensemble-based method that averages over multiple individual models and chemicals. This trend of a narrower range of predicted PODs was also observed in a previous QSAR modeling effort.^4^

Additionally, like most QSAR models, our models are only applicable to single organic compounds of small to medium sizes; mixtures, large biomolecules, polymeric chains, nanomaterials, and inorganic compounds are outside the applicability domain of OPERA 2.9.^9,10^ Different types of prediction models need to be developed for these chemicals. Additionally, our models were limited by the broad categorization of health effects.^8^ This categorization was necessitated by data availability; predicting PODs at a higher resolution, such as for specific critical effects or organ systems, would have further fragmented an already limited data set. Our models also focused on the oral exposure route, and future work is needed to incorporate additional exposure routes. Finally, our model uncertainty estimates are based on cross-validation generalization error, and future work could more fully characterize model uncertainty, for instance, at the level of individual prediction.

Overall, this study predicted *in vivo* noncancer PODs for organic chemicals, with typical RMSEs of less than 1 order of magnitude, based on structure alone. Our framework offers a high-throughput alternative to augment approaches that are based directly on *in vivo* data. Moreover, our model also conforms well to OECD guidance for evaluating QSAR models,^14^ increasing confidence in our model predictions. These predictions can, in turn, be directly used for a range of hazard, risk, and impact characterization applications, including (but not limited to) deriving probabilistic toxicity values,^39,42^ emergency response, contaminated site remediation, LCIA, CAA, and comparative risk screening. Thus, predictions from our model can substantially expand the coverage of chemicals that can be evaluated for their human health risks and impacts and thereby better promote a safer and more resilient, sustainable, and healthy environment.

## Supporting Information Available

Supplemental methods including feature preprocessing steps, model training steps (Figure S1), model performance metrics and evaluation (Figure S2), model descriptions (Table S1), and uncertainty analysis, as well as supplemental results (Figures S3–S14 and Table S2) (PDF)
Features used to the train the QSAR models for predicting points of departure (Tables S3–S4) (XLSX)
