# Supplementary material for: Two-Stage Machine Learning-Based Approach to Predict Points of Departure for Human Noncancer and Developmental/Reproductive Effects
Source: Environ Sci Technol. 2024 May 2;58(35):15638–49. doi: 10.1021/acs.est.4c00172 (PMC11371525; doi:10.1021/acs.est.4c00172)
Supplement: Supplementary file 1 — es4c00172_si_001.pdf [file es4c00172_si_001.pdf]

Supporting Information:

Two-Stage Machine Learning-Based Approach to Predict Points of Departure for Human Non-cancer and Developmental/Reproductive Effects

Jacob Kvasnicka[1], Nicolò Aurisano[2], Kerstin von Borries[2], En-Hsuan Lu[1], Peter Fantke[2], Olivier Jolliet[2], Fred A. Wright[3], Weihsueh A. Chiu*[1]

[1] Department of Veterinary Physiology and Pharmacology, Interdisciplinary Faculty of Toxicology, Texas A&M University, College Station, Texas, 77843 United States

[2] Quantitative Sustainability Assessment, Department of Environmental and Resource Engineering, Technical University of Denmark, Bygningstorvet 115, 2800 Kgs. Lyngby, Denmark

[3] Departments of Statistics and Biological Sciences and Bioinformatics Research Center, North Carolina State University, Raleigh, North Carolina, 27695 United States

*Corresponding author: Weihsueh A. Chiu, wchiu@tamu.edu

Number of pages: 26 (including cover page)

Number of Supplemental Figures: 14

Number of Supplemental Tables: 4 (2 in this file, 2 as separate supplemental Excel files)

SUPPLEMENTAL METHODS 4

Feature Preprocessing Steps 4

Model Training Steps.. 5

Figure S1. Overview of model training with feature selection. 6

Model Performance Metrics 7

Figure S2. Overview of model evaluation. 9

Table S1. Descriptions of models included in the sensitivity analysis. 10

Margin of Exposure Uncertainty Analysis 11

SUPPLEMENTAL RESULTS 12

Figure S3. Distributions of raw OPERA 2.9 features 12

Figure S4. Proportions of data completeness for OPERA 2.9 features 13

Figure S5. Model performance benchmarking. 14

Figure S6. Frequency of features deemed important across replicate models.................... 15

Figure S7. Feature importance scores for the final model for general noncancer effects.... 16

Figure S8. Feature importance scores for the final model for reproductive/developmental effects. 17

Figure S9. Feature importance scores for the replicate models for general noncancer effects. 18

Figure S10. Feature importance scores for the replicate models for reproductive/developmental effects. 19

Figure S11. Cumulative distributions of point of departure across different data sources. . 20

Figure S12. Predicted points of departure with feature selection versus without feature selection for all chemicals in this study. 21

Figure S13. Pairwise scatterplots and kernel density estimate plots for selected features. . 22

Figure S14. Predicted points of departure stratified by missing features. 23

Table S2. OECD Checklist for the assessment of (Q)SAR models. 24

REFERENCES 25

SUPPLEMENTAL METHODS

Feature Preprocessing Steps

The QSAR models for predicting points of departure (PODs) consisted of a pipeline of feature preprocessing steps and a machine learning estimator (e.g., random forest) (**Figure 1B**). The following preprocessing steps were involved:

1. Remove null variance features.
2. Exclude any features with over 30% missing values.
3. Apply a power transform to continuous features to make them more Gaussian-like. The Yeo-Johnson transform was applied, supporting both positive and negative values.[1]
4. Impute missing values with the median for a given feature.
5. Center and scale continuous features using **Equation S1**. The median and median absolute deviation (MAD) were used for robustness to potential outliers. Given a continuous feature $\overrightarrow{x^n}$ with *I* samples $x_1^n,\ x_2^n,\ \dots,\ x_i^n$:

$$x_i^{n\prime} = \frac{x_i^n - Median(\overrightarrow{x_n})}{MAD(\overrightarrow{x_n})} \qquad \text{(S1)}$$

Where:

- x_i^n is the original value of the *i*th sample for the *n*th feature.
- $x_i^{n\prime}$ is the centered and scaled value.

Note that centering and scaling do not affect decision-tree-based methods, such as Random Forest, but such regularization may improve performance for other regression-based methods.

Model Training Steps

The general training steps for model training are listed below, followed by the specific parameters used. **Figure S1** illustrates the training steps and includes pseudocode representing the algorithm:

- For each repetition *r* in {1, 2 …, *R*} and each fold *k* in {1, 2 …, *K*} folds:
  - Split the full dataset into 1 test set, *k*, with the remaining data as the training set.
  - Train a baseline model *m* on the training set.
  - Evaluate *m* on the test set using the root-mean-squared-error (RMSE) as the reference score *s*.
  - For each feature *n* and permutation repetition *p* in {1, 2, …, *P*}:
    - Permute the feature values.
    - Evaluate the model with the permuted feature.
    - Compute the RMSE score for the permuted model $s_{r,k,p}^{n}$.
    - Compute the raw importance score $i_{r,k,p}^{n}$ for feature *n* using **Equation S2:**

$$i_{r,k,p}^{n} = s - s_{r,k,p}^{n} \tag{S2}$$

- Aggregate $i_{r,k,p}^{n}$ across all repetitions *R*, folds *K*, and permutations *P* to form a vector $\vec{\iota_n}$
- Select the top 10 features with the largest median of their respective $\vec{\iota_n}$
- Train the final model using all samples and the top 10 features.

Parameters used: *K* = 5 (empirically shown to yield balanced bias-variance test error rate estimates),[2] *R* = 50 and *P* = 5.

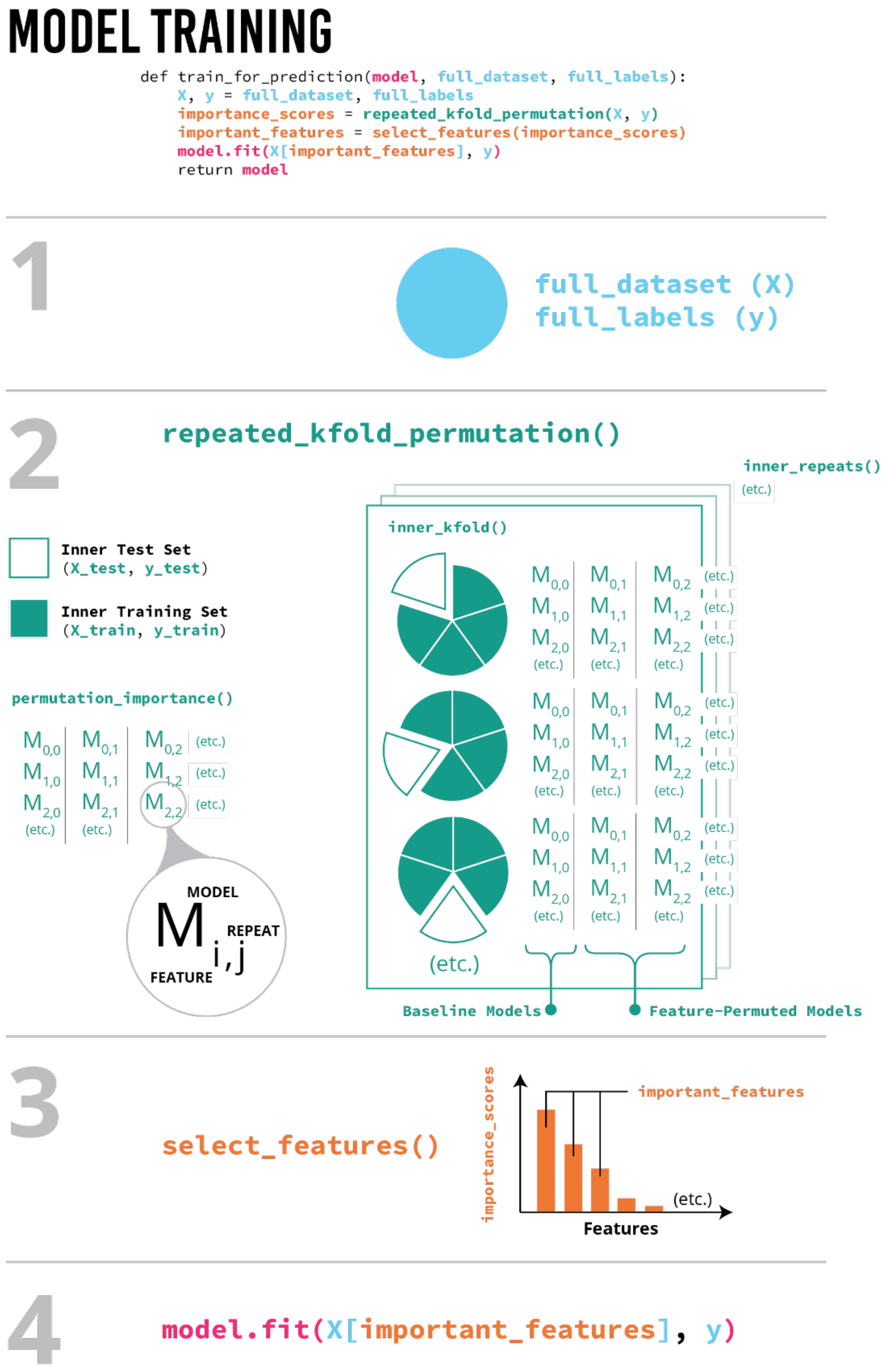

Figure S1. Overview of model training with feature selection. The top panel shows pseudocode representing the algorithm in Python. The corresponding components are illustrated in Panels 1-4. The general training steps are listed in the section above (*Model Training Steps*).

Model Performance Metrics

Figure S2 illustrates the model evaluation scheme and includes pseudocode representing the algorithm. To quantify performance, we used the root-mean-squared error (RMSE), median absolute error (MedAE), and coefficient of determination (R^2). RMSE is conceptually like a standard deviation with respect to the prediction errors:

$$RMSE = \sqrt{MSE} \tag{S3}$$

Where:

$$MSE = \frac{1}{n}\sum_{i=1}^{n}(y_i - \hat{y_i})^2 \tag{S4}$$

- $\hat{y_i}$ is the predicted value of the *i*th sample.
- y_i is the corresponding observed (or measured) value of the *i*th sample.

MedAE is a metric that is robust to outliers:

$$MedAE = Median\ (|y_1 - \widehat{y_1}|, \dots, |y_n - \widehat{y_n}|) \tag{S5}$$

R^2 represents the proportion of variance of *Y* that has been explained by the features for a given model:

$$R^2 = 1 - \frac{\sum_{i=1}^{n}(y_i - \hat{y}_i)^2}{\sum_{i=1}^{n}(y_i - \bar{y})^2} \tag{S6}$$

Where:

- $\bar{y}$ is the arithmetic mean of the observed (or measured) values.

The best possible R^2 score is 1, and a score of 0 would correspond to a constant model that always predicts the expected (average) value of *Y*. Note that in this formulation (unlike for linear regression), R^2 can also be *negative* if a model were worse than the constant model.

MODEL EVALUATION

```
def estimate_generalization_error(model, full_dataset, full_labels):
    performances = []  # initialize
    # Outer cross validation (repeated k-fold)
    for repeat in outer_repeats():
        for train_ix, test_ix in outer_kfold(repeat):
            X_train, X_test = full_dataset[train_ix], full_dataset[test_ix]
            y_train, y_test = full_labels[train_ix], full_labels[test_ix]
            importance_scores = repeated_kfold_permutation(X_train, y_train)
            important_features = select_features(importance_scores)
            model.fit(X_train[important_features], y_train)
            y_pred = model.predict(X_test[important_features])
            performance = evaluate_performance(y_test, y_pred)
            performances.append(performance)
    return performances
```

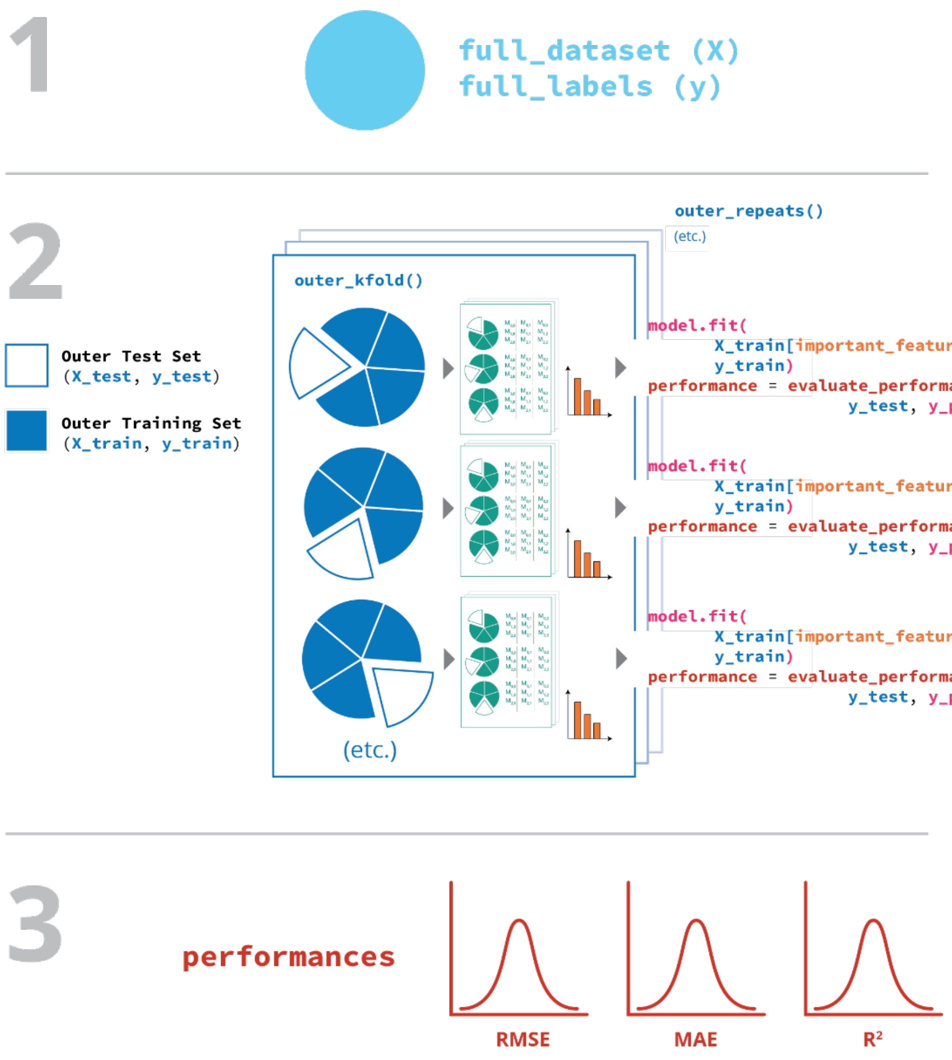

Figure S2. Overview of model evaluation. The top panel shows pseudocode representing the algorithm in Python. The corresponding components are illustrated in Panels 1-3. The performance metrics are defined in the section above (*Model Performance Metrics*). **Figure S1** shows an overview of model training with feature selection.

Table S1. Descriptions of models included in the sensitivity analysis.

Model	Description
Random Forest Regressor	Same as the final model but without feature selection, to test the hypothesis that 10 features were sufficient.
Alternative Machine Learning Estimators	Used to compare performance with Random Forest Regressor:<br>a) **LinearRegression**: Ordinary least squared linear regression.<br>b) **Ridge**: Linear least squares with L2 regularization.<br>c) **Support Vector Regression**: Epsilon-Support Vector Regression.<br>d) **Gradient Boosting Regressor**: Additive model with regression trees fit on the negative gradient of the loss function.<br>e) **XGBRegressor**: Gradient boosting variant from the XGBoost library (1.7.4).[7]<br>f) **MLPRegressor**: Multi-layer Perceptron regressor, a neural network variant, optimized using the "adam" optimizer.
OPERA w/ Exp. LD50s	Used all OPERA 2.9 features, except replacing the QSAR-predicted Lethal Dose 50 values (LD50s) with experimental LD50s from acute studies in ToxValDB.[6] Specifically, we used the median value for each chemical after conversion to human-equivalent LD50s. This sensitivity analysis evaluated the robustness of using predicted LD50s, which are available for many more chemicals than experimental LD50s.
CompTox Features	Combined features from OPERA and TEST (Toxicity Estimation Software Tool) from the CompTox Chemistry Dashboard (2.1.1) by U.S. EPA.[3] These features are described in a supplemental Excel file (**Table S4**). This sensitivity analysis evaluated the impact of using readily available pre-selected/pre-calculated features instead of generating features directly from the OPERA 2.9 software. For consistency, we preprocessed these features by excluding any chemicals that had been filtered out in the QSAR-standardization workflow by Mansouri et al.[4,5]
RDKit Features	Used all two-dimensional descriptors from the RDKit Python library (2022.09.05). First, for each chemical, a molecule object was instantiated from the "QSAR-ready" SMILES string. Next, the function, "CalcDescriptors," was applied to each molecule, resulting in 208 features. Some features had null median absolute deviations and were therefore not scaled in the preprocessing pipeline described in section, "Feature Preprocessing Steps."
No Imputation	Used only samples for which all OPERA 2.9 features had no missing values, so that no imputation was performed.

The sensitivity analysis was used to assess generalization error sensitivity to different datasets, feature preprocessing, and machine learning estimators. Our baseline Final Model was described in the main text, involving feature selection among all 39 OPERA 2.9 features, imputation of missing values, and the Random Forest Regressor. All models were applied to the same chemicals, except the model involving no imputation was restricted to those chemicals with no missing feature values (n =184 – 227).

Margin of Exposure Uncertainty Analysis

We assessed the contribution of POD_{QSAR} (hazard) uncertainty to the overall uncertainty in the margin of exposure, in addition to exposure uncertainty from SEEM3 (Systematic Empirical Evaluation of Models) by U.S. EPA.[8] Specifically, we derived 90% prediction intervals of POD_{QSAR} uncertainty for each percentile of exposure uncertainty for the median individual.

For the *ith* chemical:

$$L_i \leq log_{10}MOE_i \leq U_i \quad \text{(S7)}$$

Where:

$$L_i = log_{10}MOE_i - z \times \epsilon \quad \text{(S8)}$$

$$U_i = log_{10}MOE_i + z \times \epsilon$$

Where:

- L_i is the lower bound of the prediction interval.
- U_i is the upper bound of the prediction interval.
- $log_{10}MOE_i$ is the predicted margin of exposure (**Equation 1** in main text) in log10-units.
- ϵ is the measure of POD_{QSAR} uncertainty in log10-units. In this analysis, we used the median RMSE from the cross-validation scheme described above (**Figure S3**).
- z is a z-score of 1.645 corresponding to a 90% confidence level of log10 values.

SUPPLEMENTAL RESULTS

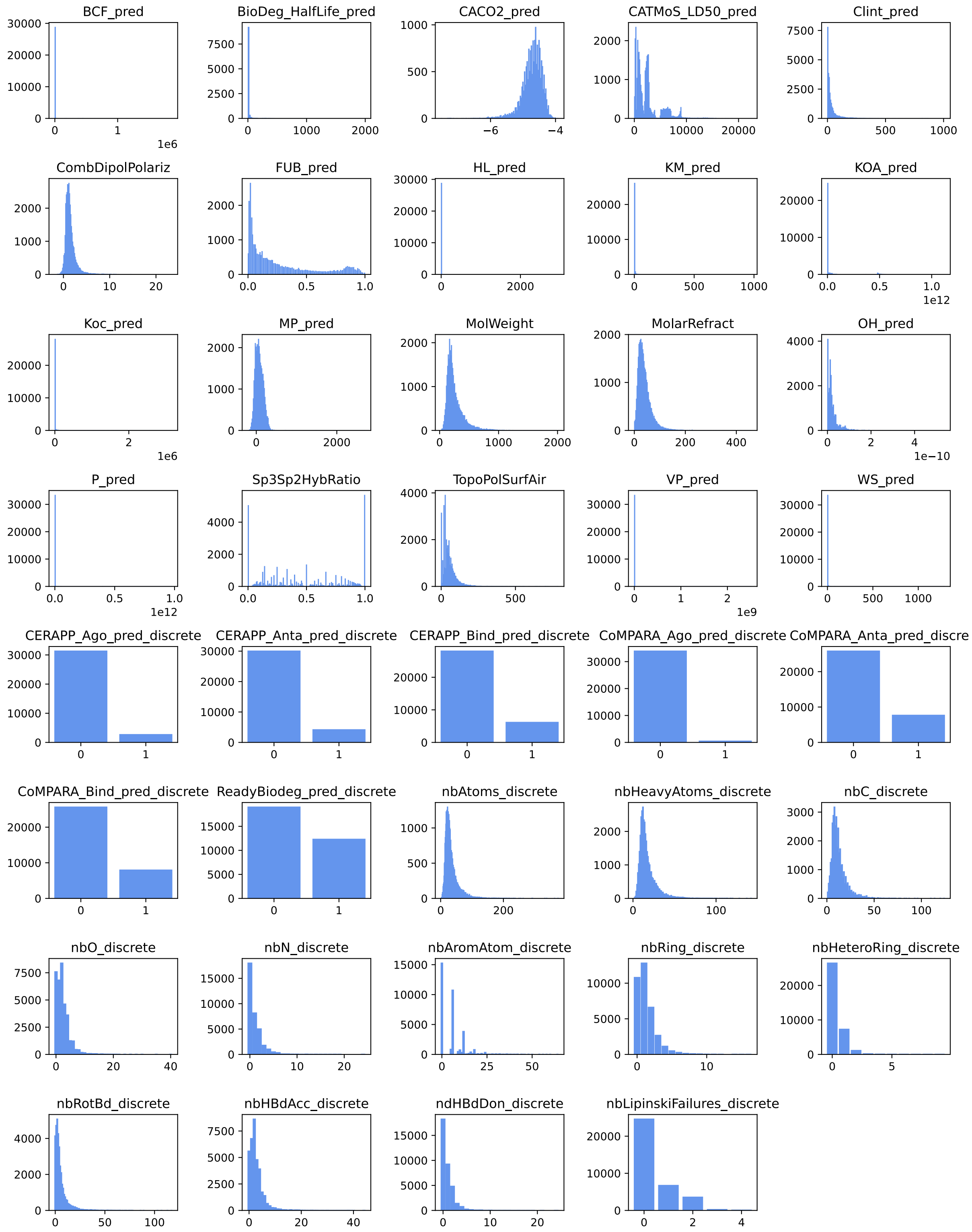

Figure S3. Distributions of raw OPERA 2.9 features.[9,10] Continuous features are represented with histograms, whereas discrete features are represented with bar plots indicating the count of samples for each unique value. The data are shown for all chemicals in this study. Feature descriptions are included in a supplemental Excel file (**Table S3**).

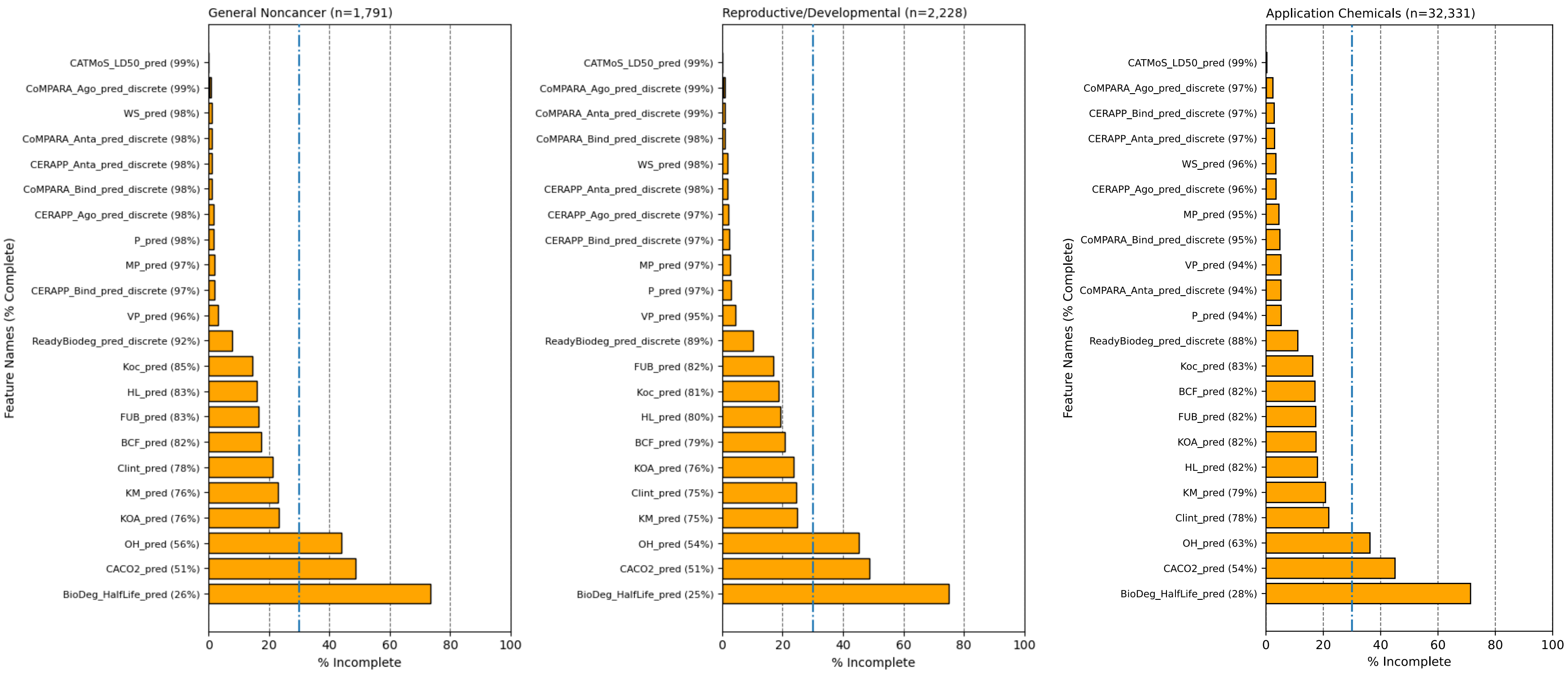

Figure S4. Proportions of data completeness for OPERA 2.9 features.[9,10] The figure is subdivided by the target effect category for training data chemicals (left and middle panels) and for the application chemicals (right panel) that were on the Merged NORMAN Suspect List (SusDat)[11,12] and within the applicability domain of SEEM3,[8] excluding any training chemicals. A vertical dashed line denotes the threshold above which features were excluded (see section, *Model Training and Evaluation*): Biodegradation half-life for compounds containing only carbon and hydrogen (BioDeg_HalfLife_pred): 74-75% missing; Caco-2 permeability (CACO2_pred): 49% missing; Rate constant for the atmospheric, gas-phase reaction with photochemically produced hydroxyl radicals (OH_pred): 44-45% missing. Features with no missing values are not shown. Feature descriptions are included in a supplemental Excel file (**Table S3**). Note: n, sample size.

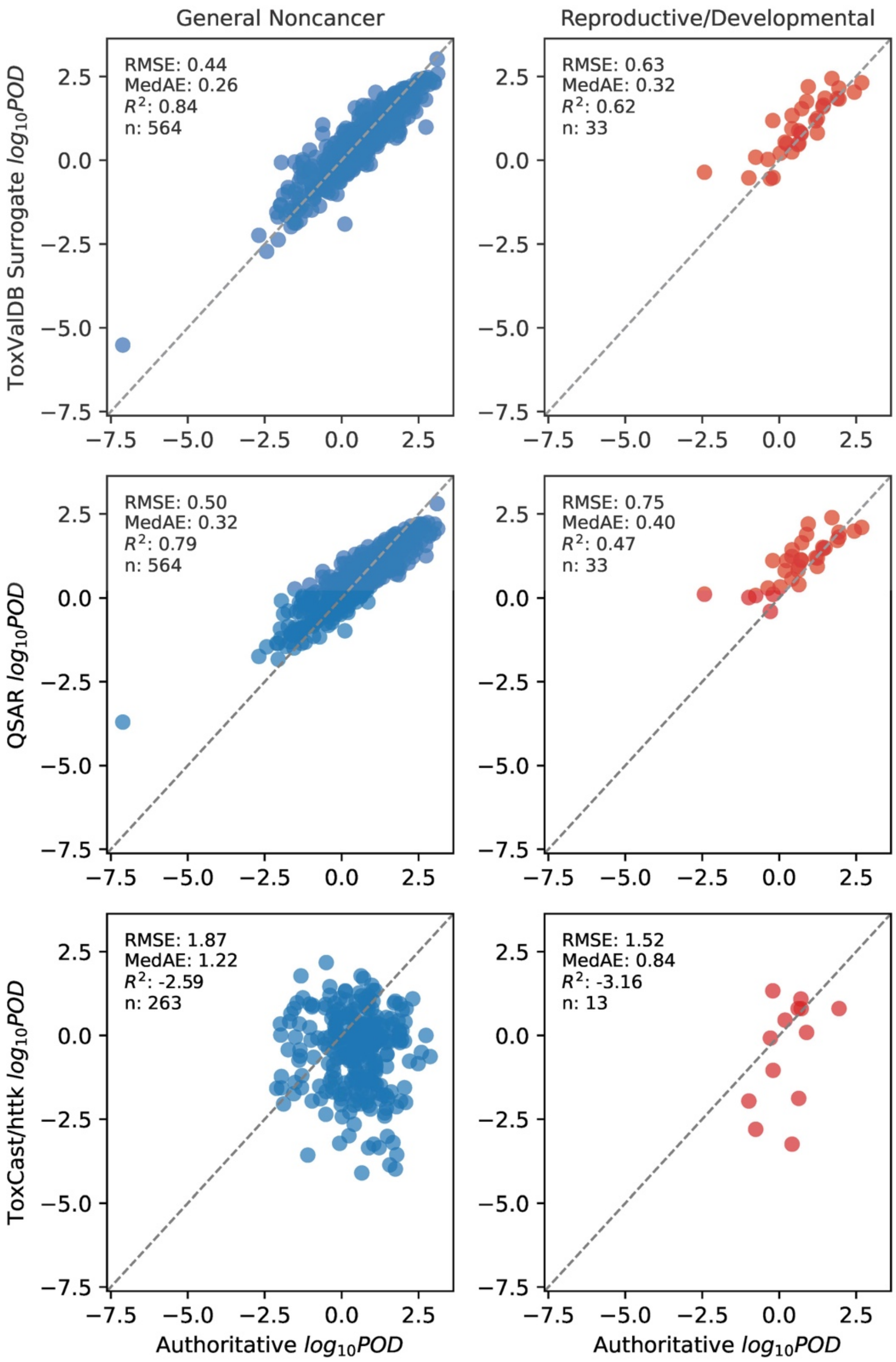

Figure S5. Model performance benchmarking. Point of departure estimates are compared against authoritative values. "ToxValDB Surrogate" refers to the surrogate values from Table S5 of Aurisano et al. (2023).[13] "QSAR" refers to the final model developed in this study, described in the main text. "ToxCast/httk" refers to the combination of high-throughput *in vitro* bioactivity data with toxicokinetic data using reverse dosimetry. Specifically, these values are the $POD_{NAM,50}$ values from Table S2 of Paul Friedman et al. (2020)[14]. The figure is further subdivided by the target effect category from left to right. Note: POD, point of departure; QSAR, Quantitative Structure-Activity Relationship; RMSE, root-mean-squared error, MedAE, median absolute error; R^2, coefficient of determination; n, sample size.

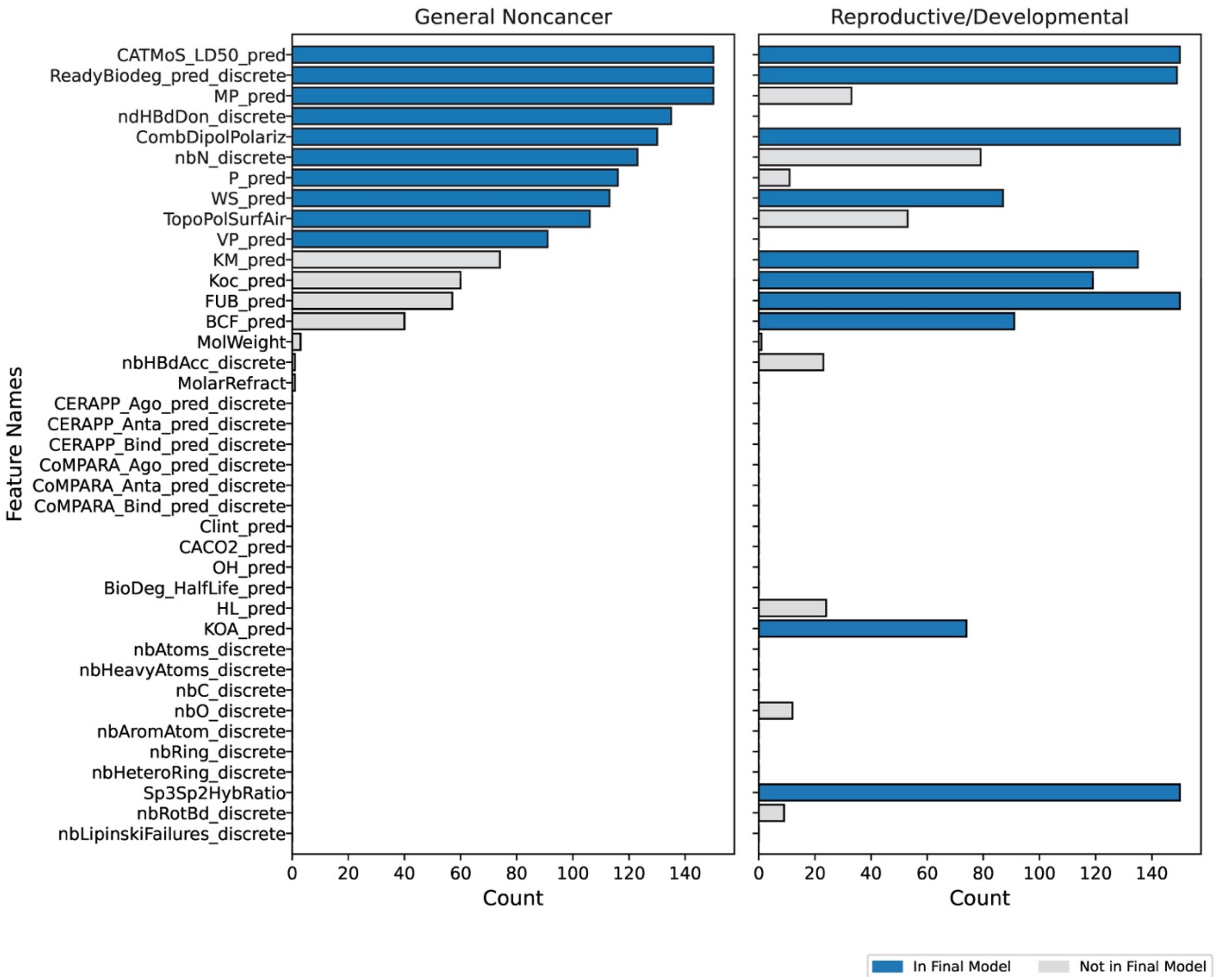

Figure S6. Frequency of features deemed important across replicate models. Features were extracted from OPERA 2.9.[9,10] The figure is subdivided by the target effect category from left to right. The x-axis represents the number of times each feature was deemed important across the cross-validated replicate models illustrated in **Figure S2**. The feature selection scheme is illustrated in **Figure S1**. Features present in the final models are highlighted with a distinct color. The remaining important features were excluded from the final models to avoid overfitting. Feature descriptions are included in a supplemental Excel file (**Table S3**).

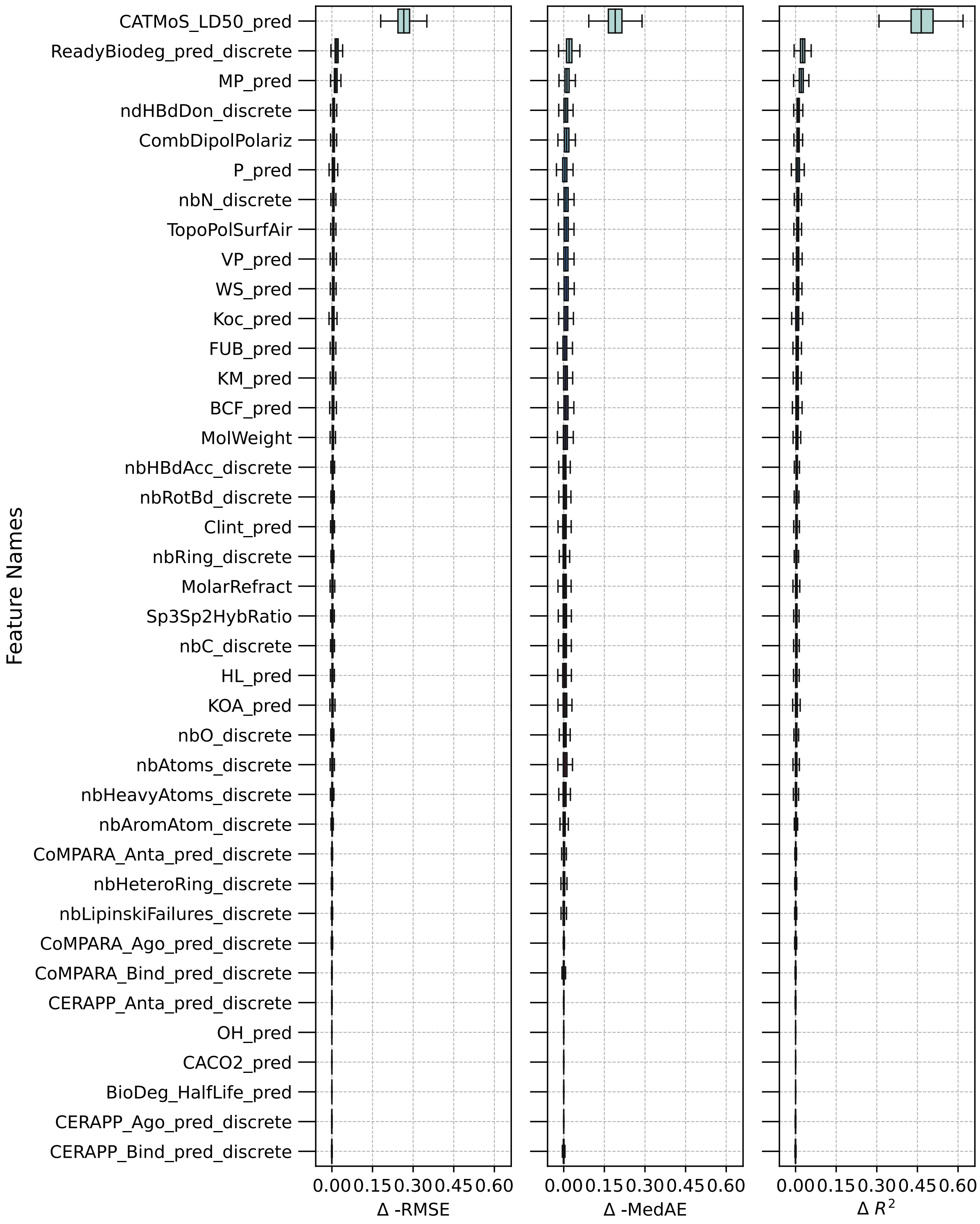

Figure S7. Feature importance scores for the final model for general noncancer effects. Features were extracted from OPERA 2.9.[9,10] These scores were used to select important features (see section, *Model Training Steps*). The feature selection scheme is illustrated in **Figure S1**. The boxes show the median and interquartile range with outliers omitted. Feature descriptions are included in a supplemental Excel file (**Table S3**). Note: RMSE, root-mean-squared error, MedAE, median absolute error; R^2, coefficient of determination.

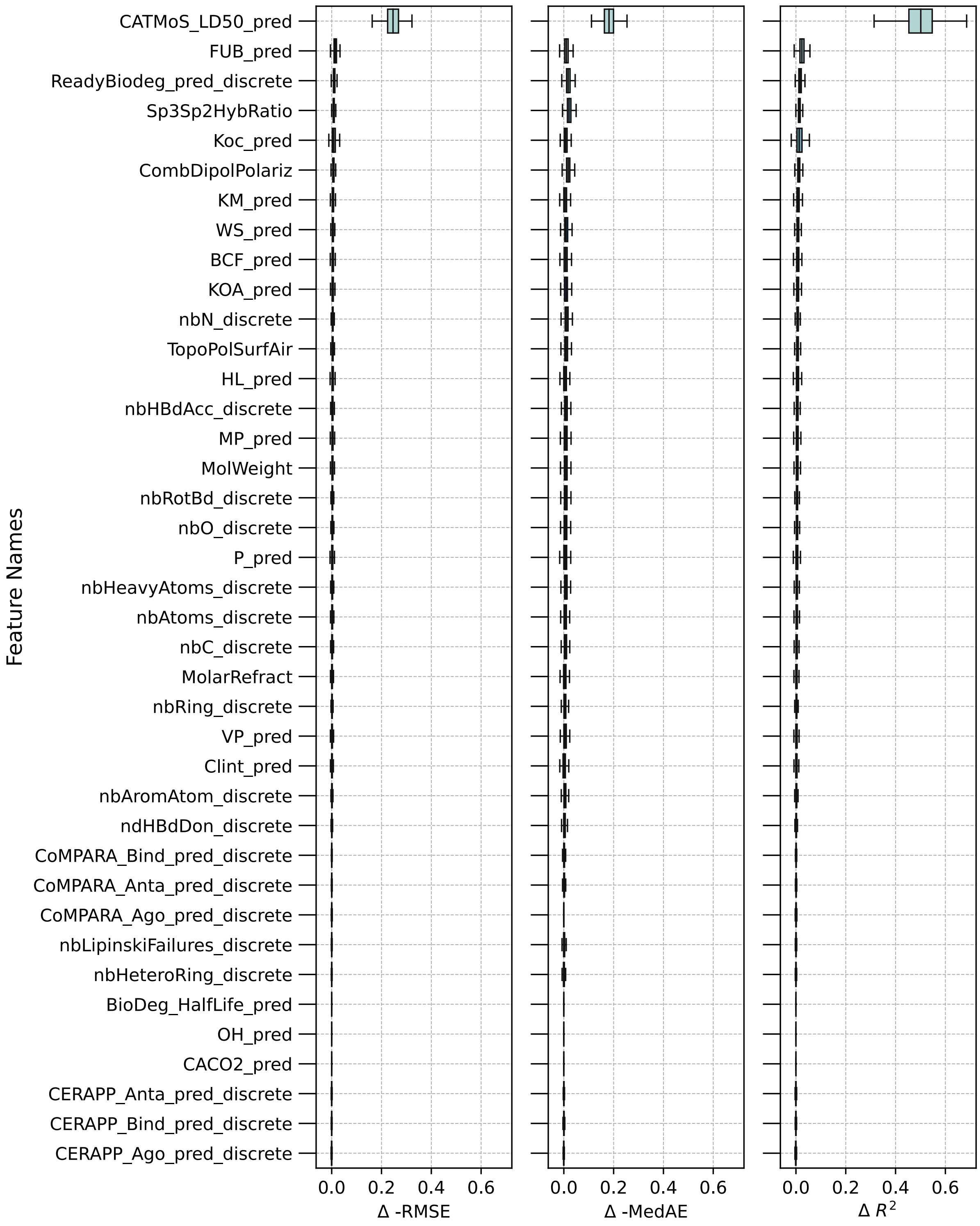

Figure S8. Feature importance scores for the final model for reproductive/developmental effects. Features were extracted from OPERA 2.9.[9,10] These scores were used to select important features (see section, *Model Training Steps*). The feature selection scheme is illustrated in **Figure S1**. The boxes show the median and interquartile range with outliers omitted. Feature descriptions are included in a supplemental Excel file (**Table S3**). Note: RMSE, root-mean-squared error, MedAE, median absolute error; R^2, coefficient of determination.

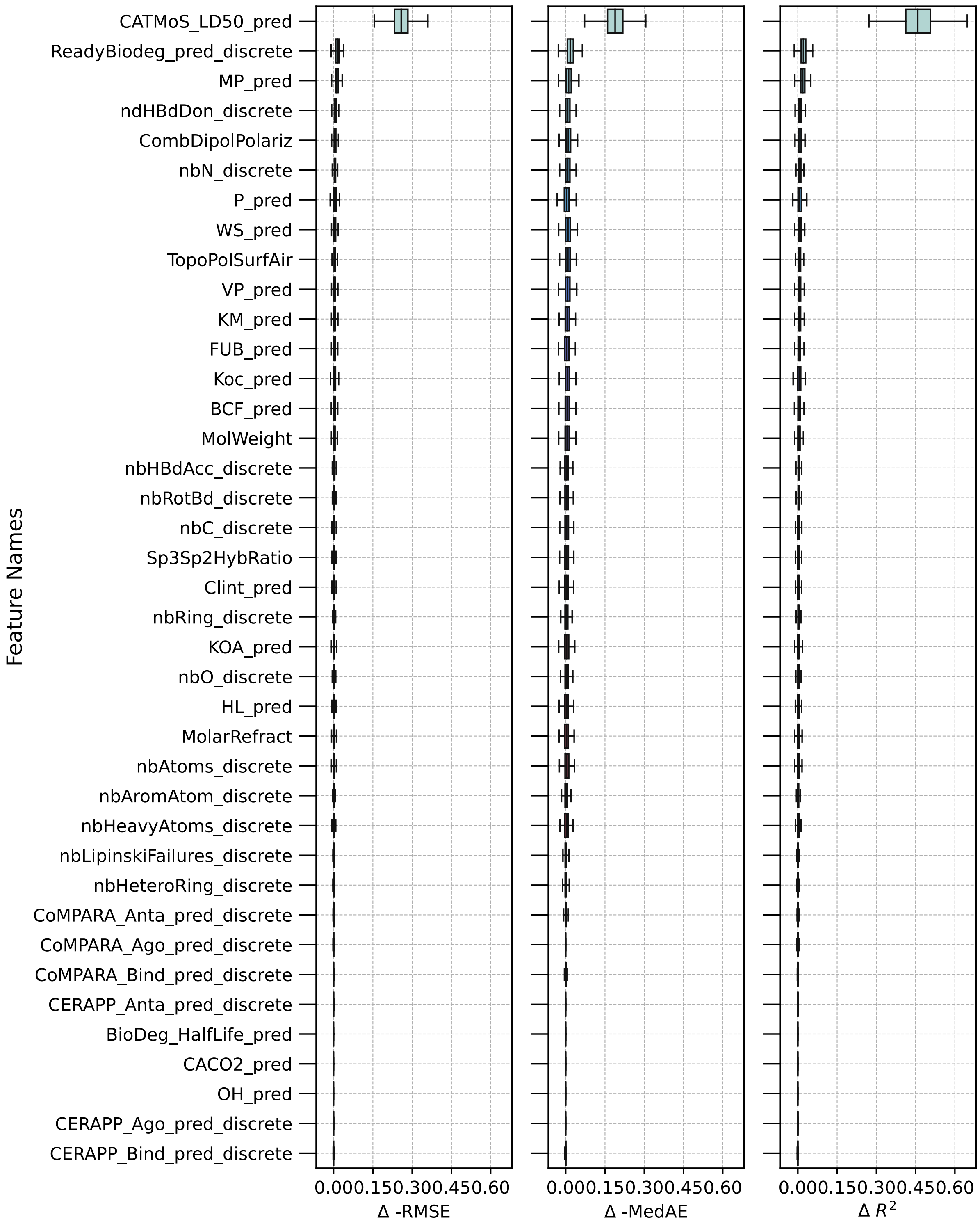

Figure S9. Feature importance scores for the replicate models for general noncancer effects. Features were extracted from OPERA 2.9.[9,10] These scores were used to select important features (see section, *Model Training Steps*). The feature selection scheme is illustrated in **Figure S1**. The boxes show the median and interquartile range with outliers omitted. Feature descriptions are included in a supplemental Excel file (**Table S3**). Note: RMSE, root-mean-squared error, MedAE, median absolute error; R^2, coefficient of determination.

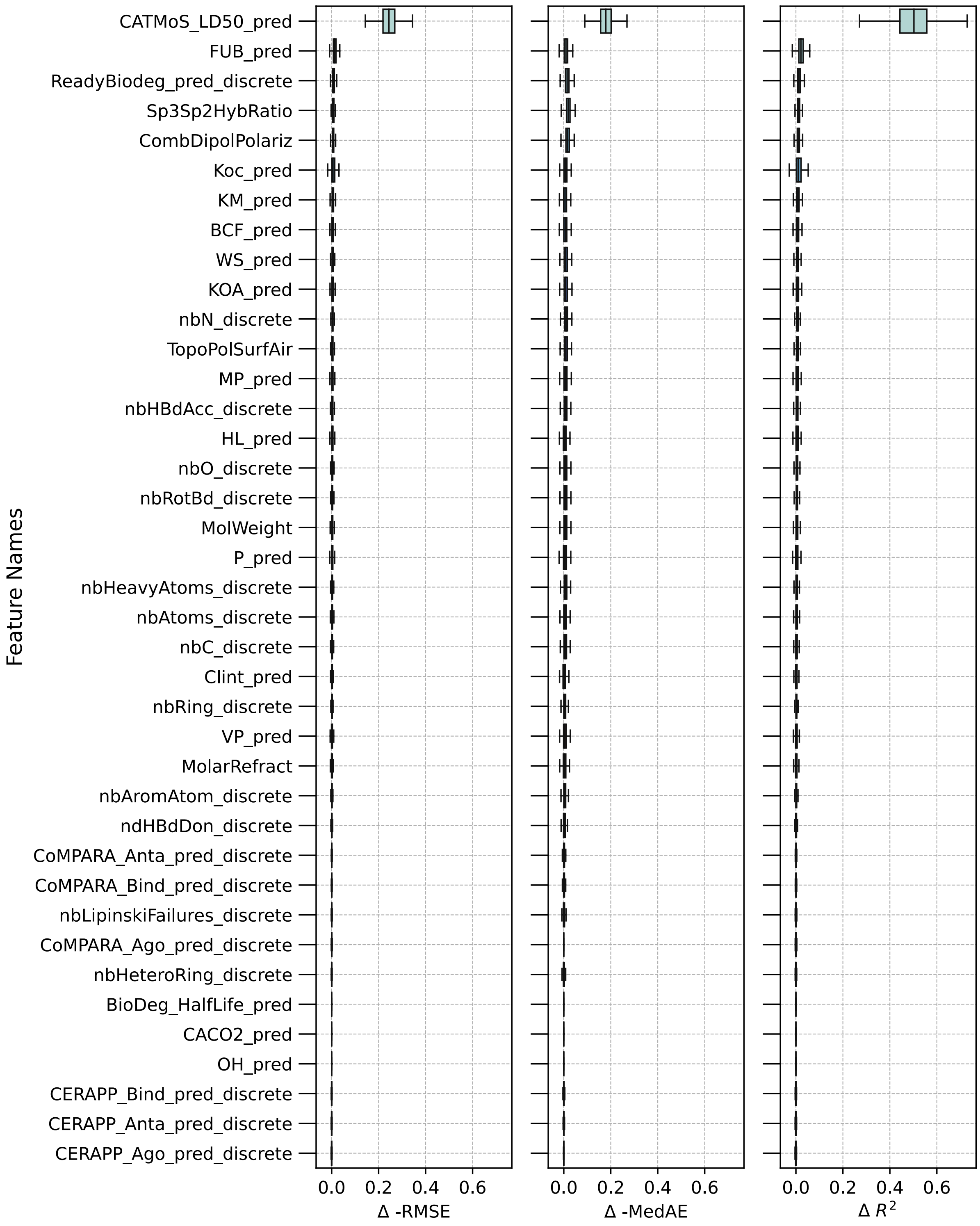

Figure S10. Feature importance scores for the replicate models for reproductive/developmental effects. Features were extracted from OPERA 2.9.[9,10] These scores were used to select important features (see section, *Model Training Steps*). The feature selection scheme is illustrated in **Figure S1**. The boxes show the median and interquartile range with outliers omitted. Feature descriptions are included in a supplemental Excel file (**Table S3**). Note: RMSE, root-mean-squared error, MedAE, median absolute error; R^2, coefficient of determination.

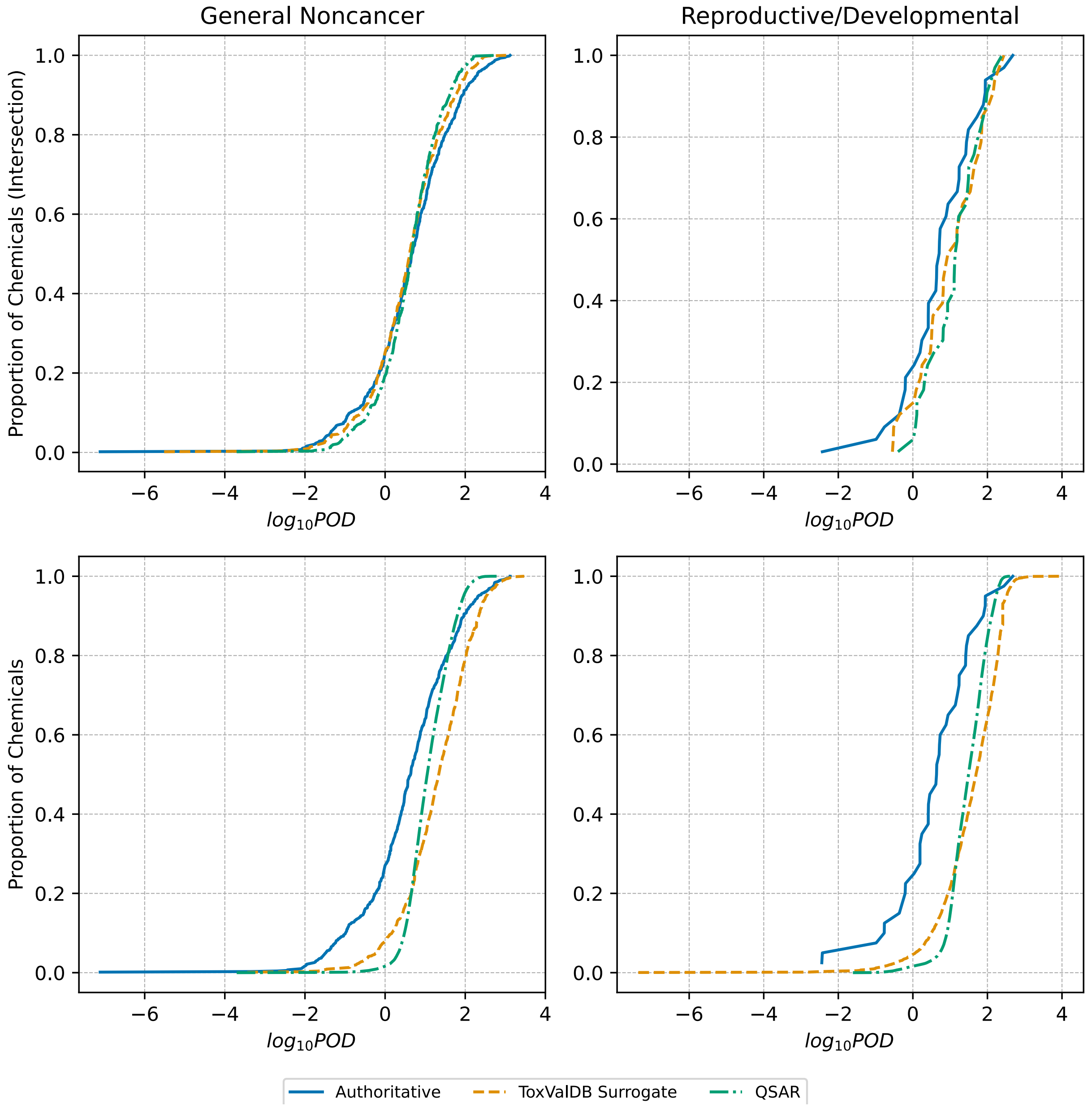

Figure S11. Cumulative distributions of point of departure across different data sources. "Authoritative" refers to the values from authoritative and regulatory assessments from Figure S5 of Aurisano et al. (2023).[13] "ToxValDB Surrogate" refers to the surrogate values from Table S5 of Aurisano et al. "QSAR" refers to the final model developed in this study, described in the main text. The intersection of chemicals is shown in the top half of the figure. The bottom half shows all chemicals with original authoritative PODs ($POD_{authoritative}$), all chemicals from ToxValDB with surrogate PODs ($POD_{surrogate}$), excluding those chemicals with $POD_{authoritative}$ values, and all "application chemicals" with QSAR-derived PODs (POD_{QSAR}), excluding those chemicals in the other two datasets. The application chemicals were on the Merged NORMAN Suspect List (SusDat)[11,12] and within the applicability domain of SEEM3.[8] The figure is further subdivided by the target effect category from left to right. Note: POD, point of departure.

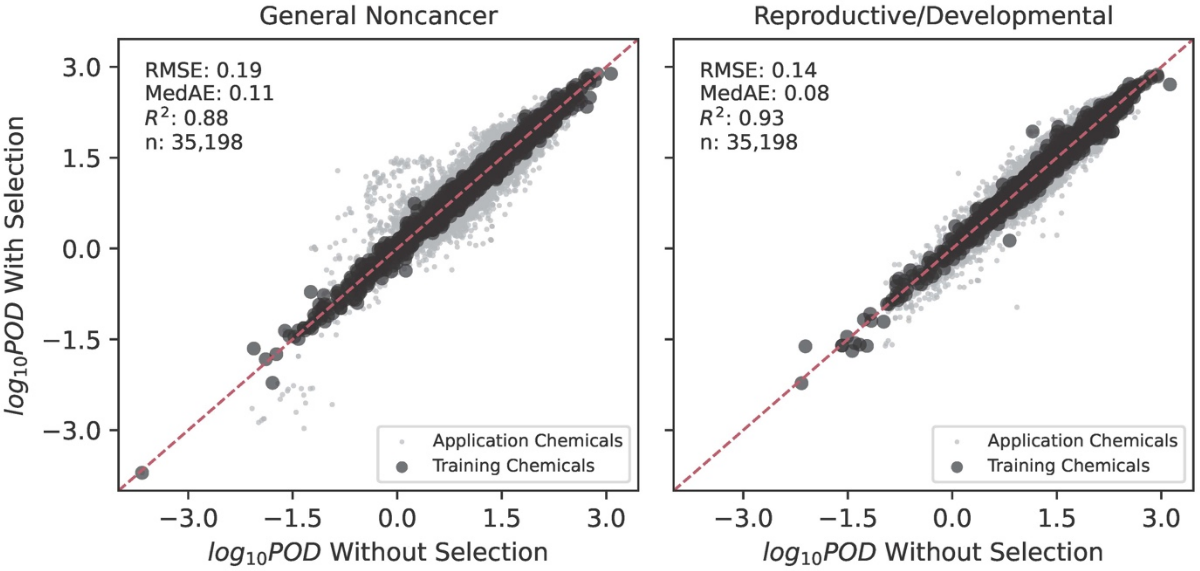

Figure S12. Predicted points of departure with feature selection versus without feature selection for all chemicals in this study. "Application chemicals" refer to those on the Merged NORMAN Suspect List (SusDat)[11,12] and within the applicability domain of SEEM3,[8] excluding any training chemicals. The figure is subdivided by the target effect category from left to right. Note: POD, point of departure; RMSE, root-mean-squared error, MedAE, median absolute error; R^2, coefficient of determination; n, sample size.

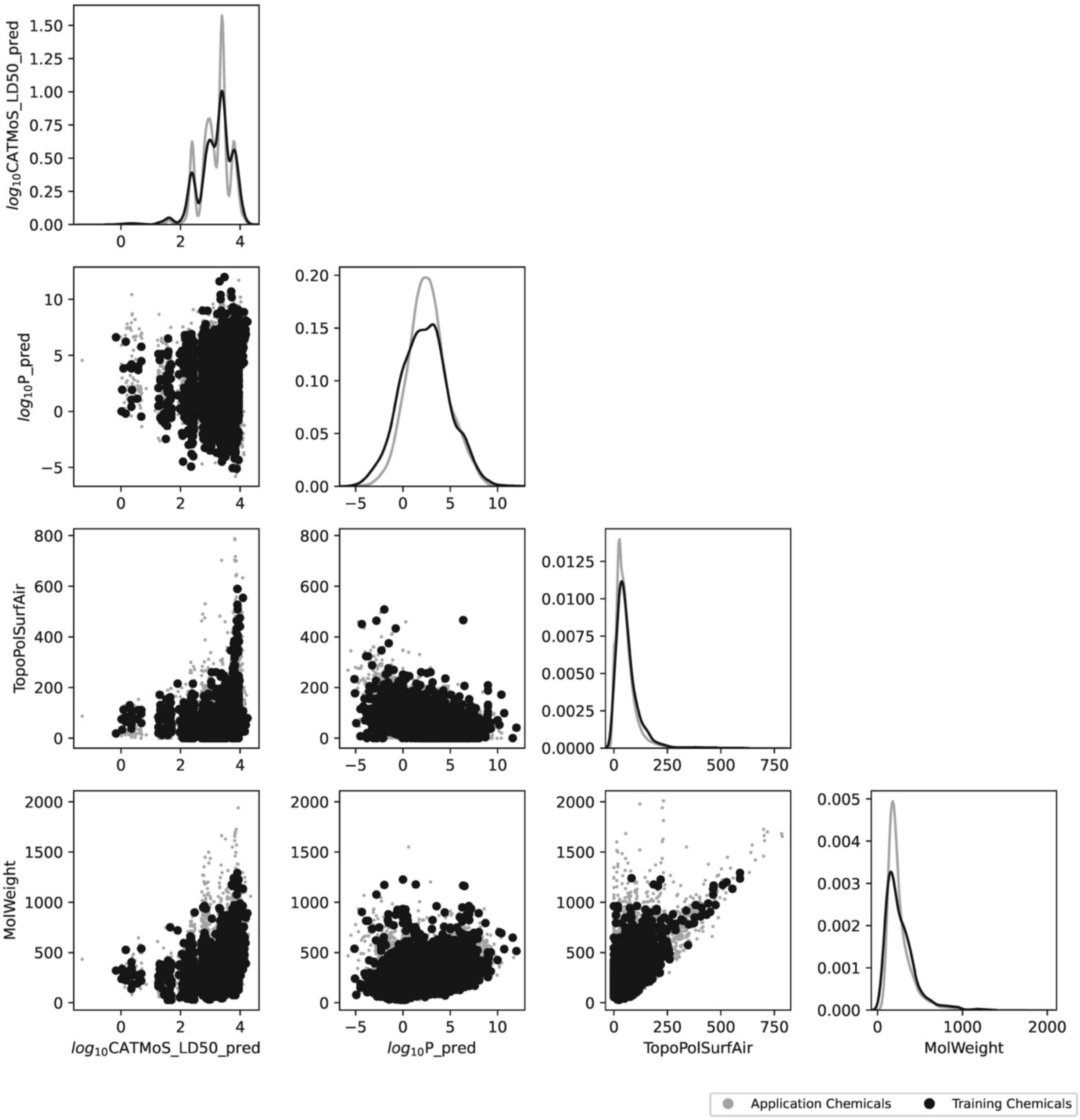

Figure S13. Pairwise scatterplots and kernel density estimate plots for selected features. Features were extracted from OPERA 2.9.[9,15,10] Diagonal cells show the kernel density estimate plots, illustrating the distribution of individual features. "Application chemicals" refer to those on the Merged NORMAN Suspect List (SusDat)[11,12] and within the applicability domain of SEEM3,[8] excluding any training chemicals. Note: log_{10}_CATMoS_LD50_pred, LD50 point estimate model from the Collaborative Acute Toxicity Modeling Suite [units of log10-transformed mg·(kg-d)$^{-1}$]; log_{10}P_pred, octanol-water partition coefficient (log10-transformed); TopoPolSurfAir, topological polar surface area (A^2); MolWeight (g·mole^{-1})

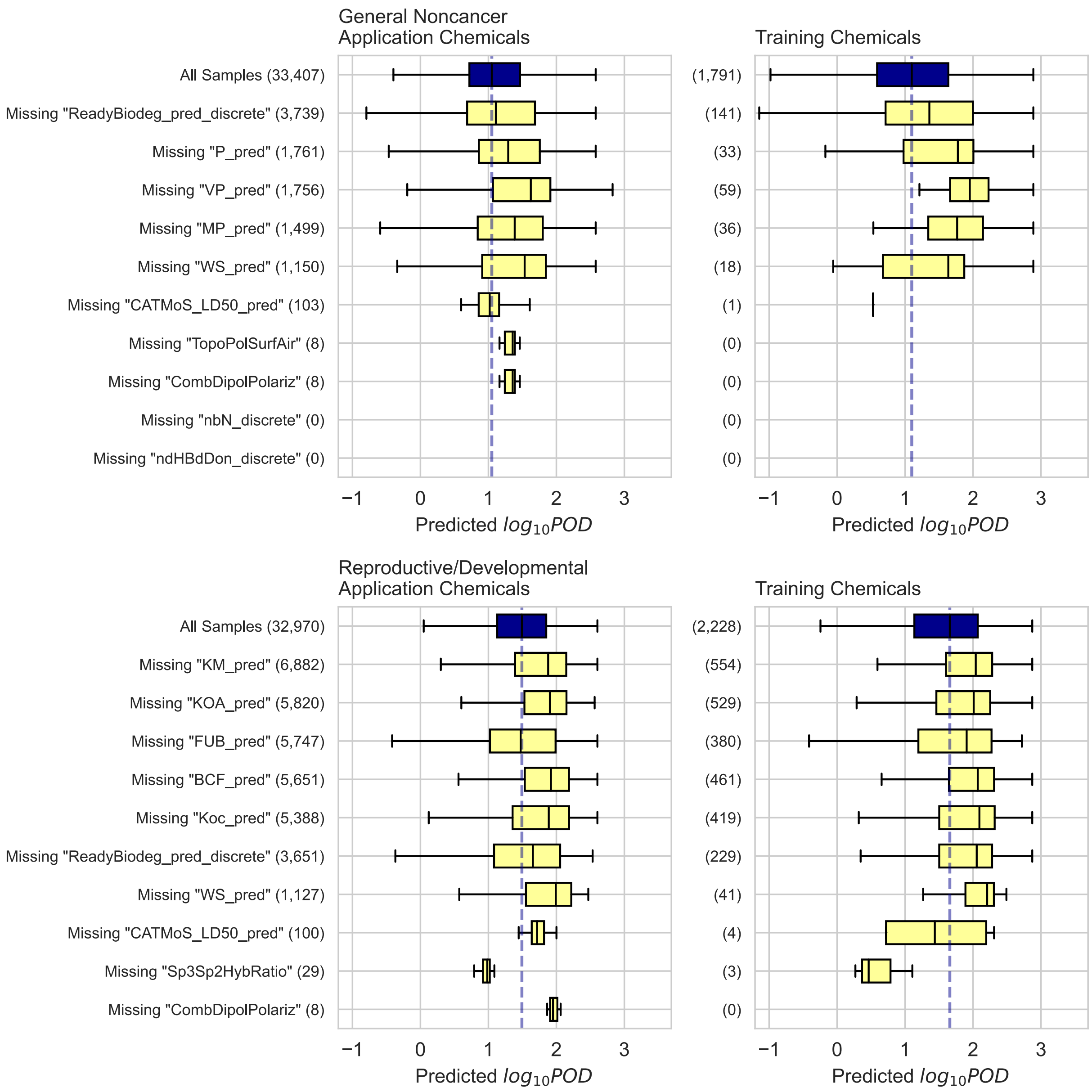

Figure S14. Predicted points of departure stratified by missing features. Feature names are displayed in the y-axis with sample sizes in parentheses. A vertical dashed line passes through the median of "All Samples." "Application chemicals" refer to those on the Merged NORMAN Suspect List (SusDat)[11,12] and within the applicability domain of SEEM3,[8] excluding any training chemicals. The figure is further subdivided by the target effect category from top to bottom. The boxes show the median and interquartile range with outliers omitted. Feature descriptions are included in a supplemental Excel file (**Table S3**). Note: POD, point of departure.

Table S2. OECD Checklist for the assessment of (Q)SAR models.

Principle	Assessment element	Outcome
**Defined endpoint**		
1.1	Clear scientific and regulatory purpose	The predicted endpoint is a human-equivalent point of departure (POD) in mg·(kg-d)$^{-1}$ for oral exposure, to be used as a surrogate for *in vivo* PODs based on experimental animal studies. Separate predictions are made for general noncancer effects and reproductive/developmental effects.
1.2	Transparency of the underlying experimental data	The underlying experimental data are from the ToxValDB and were previously reported in Aurisano et al. (2023).[13] References to the underlying toxicology studies are in the ToxValDB Version 9.1 available from U.S. EPA.[6]
1.3	Quality of the underlying experimental data	Original data curation was performed by U.S. EPA. Additional curation procedure was described by Aurisano et al., 2023.[13] For the QSAR model, chemicals with ≤ 3 studies were excluded to ensure higher quality (**Figure 1**).
**Unambiguous algorithm**		
2.1	Description of the algorithm and/or software	The paper describes the use of a random forests implementation in scikit-learn,[16] referencing the specific version number (1.2.2). The methods for feature selection and cross validation are generally described in the text and **Figures S1-S2**. OPERA 2.9, used for feature extraction, is available publicly on GitHub with a user guide.[10]
2.2	Inputs and other options	Current model outputs are available for > 30,000 chemicals expected to occur in the environment[11,12] that are within the applicability domain of SEEM3,[8] based on input of chemical identifiers (DTXSID) (**Figure 4**). A graphical user interface will be made available for downloading predictions.
2.3	Model accessibility	The model will be publicly available upon publication.
**Defined domain of applicability**		
3.1	Clear definition of the applicability domain and limitations of the model	The paper defines the applicability domain (AD) in two stages. First, chemicals must pass the "QSAR-ready" standardization workflow in order to be considered within the "general AD" of the model. Second, feature-specific ADs are generated from OPERA 2.9.[9,10] Predictions can still be generated for chemicals outside this feature-specific AD with median imputation, but those features are flagged.
**Appropriate measures of goodness-of-fit, robustness and predictivity**		
4.1	Goodness-of-fit, robustness	The paper transparently describes the statistical metrics and cross-validation method used to evaluate performance (**Equations S3-S6** and **Figure S2**).
4.2	Predictivity	Extensive, two-stage cross-validation was used to estimate the predictive power (R^2) and prediction errors (RMSE, MedAE) for an external dataset (**Figure S2**). Performance was also evaluated relative to reference values in the form of authoritative PODs (**Figure S5**).
**Mechanistic interpretation**		
5.1	Plausibility of the mechanistic interpretation	The most important feature is consistently the QSAR-predicted LD50, derived from *in vivo* rat acute oral toxicity studies,[15] which is an indicator of the acute mammalian potency of a chemical. Other important features are physical and chemical properties with clear interpretations (**Figures S6-S10**). Feature descriptions are included in a supplemental Excel file (**Tables S3-S4**).

Results of applying the *(Q)SAR Assessment Framework* to our modeling framework (**Figure 1**), demonstrating how our framework conforms to general principles and criteria for use of QSAR models.[17]

REFERENCES

(1) Yeo, I.-K.; Johnson, R. A. A New Family of Power Transformations to Improve Normality or Symmetry. *Biometrika* **2000**, *87* (4), 954–959.

(2) Hastie, T.; Tibshirani, R.; Friedman, J. *The Elements of Statistical Learning*; Springer Series in Statistics; Springer: New York, NY, 2009. https://doi.org/10.1007/978-0-387-84858-7.

(3) Williams, A. J.; Grulke, C. M.; Edwards, J.; McEachran, A. D.; Mansouri, K.; Baker, N. C.; Patlewicz, G.; Shah, I.; Wambaugh, J. F.; Judson, R. S.; Richard, A. M. The CompTox Chemistry Dashboard: A Community Data Resource for Environmental Chemistry. *J. Cheminformatics* **2017**, *9* (1), 61. https://doi.org/10.1186/s13321-017-0247-6.

(4) Mansouri, K.; Abdelaziz, A.; Rybacka, A.; Roncaglioni, A.; Tropsha, A.; Varnek, A.; Zakharov, A.; Worth, A.; Richard, A. M.; Grulke, C. M.; Trisciuzzi, D.; Fourches, D.; Horvath, D.; Benfenati, E.; Muratov, E.; Wedebye, E. B.; Grisoni, F.; Mangiatordi, G. F.; Incisivo, G. M.; Hong, H.; Ng, H. W.; Tetko, I. V.; Balabin, I.; Kancherla, J.; Shen, J.; Burton, J.; Nicklaus, M.; Cassotti, M.; Nikolov, N. G.; Nicolotti, O.; Andersson, P. L.; Zang, Q.; Politi, R.; Beger, R. D.; Todeschini, R.; Huang, R.; Farag, S.; Rosenberg, S. A.; Slavov, S.; Hu, X.; Judson, R. S. CERAPP: Collaborative Estrogen Receptor Activity Prediction Project. *Environ. Health Perspect.* **2016**, *124* (7), 1023–1033. https://doi.org/10.1289/ehp.1510267.

(5) Mansouri, K. QSAR-Ready, 2022. https://github.com/NIEHS/QSAR-ready (accessed 2023-11-17).

(6) Judson, R. ToxValDB: Compiling Publicly Available In Vivo Toxicity Data, 2018. https://www.epa.gov/sites/production/files/2018-12/documents/comptox_cop_dec_20_2018_final.pdf (accessed 2023-11-16).

(7) Chen, T.; Guestrin, C. XGBoost: A Scalable Tree Boosting System. In *Proceedings of the 22nd ACM SIGKDD International Conference on Knowledge Discovery and Data Mining*; ACM: San Francisco California USA, 2016; pp 785–794. https://doi.org/10.1145/2939672.2939785.

(8) Ring, C. L.; Arnot, J. A.; Bennett, D. H.; Egeghy, P. P.; Fantke, P.; Huang, L.; Isaacs, K. K.; Jolliet, O.; Phillips, K. A.; Price, P. S.; Shin, H.-M.; Westgate, J. N.; Setzer, R. W.; Wambaugh, J. F. Consensus Modeling of Median Chemical Intake for the U.S. Population Based on Predictions of Exposure Pathways. *Environ. Sci. Technol.* **2019**, *53* (2), 719–732. https://doi.org/10.1021/acs.est.8b04056.

(9) Mansouri, K.; Grulke, C. M.; Judson, R. S.; Williams, A. J. OPERA Models for Predicting Physicochemical Properties and Environmental Fate Endpoints. *J. Cheminformatics* **2018**, *10* (1), 10. https://doi.org/10.1186/s13321-018-0263-1.

(10) Mansouri, K. OPERA, 2023. https://github.com/NIEHS/OPERA (accessed 2023-11-17).

(11) Mohammed Taha, H.; Aalizadeh, R.; Alygizakis, N.; Antignac, J.-P.; Arp, H. P. H.; Bade, R.; Baker, N.; Belova, L.; Bijlsma, L.; Bolton, E. E.; Brack, W.; Celma, A.; Chen, W.-L.; Cheng, T.; Chirsir, P.; Čirka, Ľ.; D'Agostino, L. A.; Djoumbou Feunang, Y.; Dulio, V.; Fischer, S.; Gago-Ferrero, P.; Galani, A.; Geueke, B.; Głowacka, N.; Glüge, J.; Groh, K.; Grosse, S.; Haglund, P.; Hakkinen, P. J.; Hale, S. E.; Hernandez, F.; Janssen, E. M.-L.; Jonkers, T.; Kiefer, K.; Kirchner, M.; Koschorreck, J.; Krauss, M.; Krier, J.; Lamoree, M. H.; Letzel, M.; Letzel, T.; Li, Q.; Little, J.; Liu, Y.; Lunderberg, D. M.; Martin, J. W.; McEachran, A. D.; McLean, J. A.; Meier, C.; Meijer, J.; Menger, F.; Merino, C.; Muncke, J.; Muschket, M.; Neumann, M.; Neveu, V.; Ng, K.; Oberacher, H.; O'Brien, J.; Oswald, P.; Oswaldova, M.; Picache, J. A.; Postigo, C.; Ramirez, N.; Reemtsma, T.; Renaud, J.; Rostkowski, P.; Rüdel, H.; Salek, R. M.; Samanipour, S.; Scheringer, M.; Schliebner, I.; Schulz, W.; Schulze, T.; Sengl, M.; Shoemaker, B. A.; Sims, K.; Singer, H.; Singh, R. R.; Sumarah, M.; Thiessen, P. A.; Thomas, K. V.; Torres, S.; Trier, X.; Van Wezel, A. P.; Vermeulen, R. C. H.; Vlaanderen, J. J.; Von Der Ohe, P. C.; Wang, Z.; Williams, A. J.; Willighagen, E. L.; Wishart, D. S.; Zhang, J.; Thomaidis, N. S.; Hollender, J.; Slobodnik, J.; Schymanski, E. L. The NORMAN Suspect List Exchange (NORMAN-SLE): Facilitating European and Worldwide Collaboration on Suspect Screening in High Resolution Mass Spectrometry. *Environ. Sci. Eur.* **2022**, *34* (1), 104. https://doi.org/10.1186/s12302-022-00680-6.

(12) NORMAN Network; Aalizadeh, R.; Alygizakis, N.; Schymanski, E.; Slobodnik, J.; Fischer, S.; Cirka, L. S0 | SUSDAT | Merged NORMAN Suspect List: SusDat (NORMAN-SLE-S0.0.4.3) [Data Set]. Zenodo. Https://Doi.Org/10.5281/Zenodo.6853705, 2022.

(13) Aurisano, N.; Jolliet, O.; Chiu, W. A.; Judson, R.; Jang, S.; Unnikrishnan, A.; Kosnik, M. B.; Fantke, P. Probabilistic Points of Departure and Reference Doses for Characterizing Human Noncancer and Developmental/Reproductive Effects for 10,145 Chemicals. *Environ. Health Perspect.* **2023**, *131* (3), 037016. https://doi.org/10.1289/EHP11524.

(14) Paul Friedman, K.; Gagne, M.; Loo, L.-H.; Karamertzanis, P.; Netzeva, T.; Sobanski, T.; Franzosa, J. A.; Richard, A. M.; Lougee, R. R.; Gissi, A. Utility of in Vitro Bioactivity as a Lower Bound Estimate of in Vivo Adverse Effect Levels and in Risk-Based Prioritization. *Toxicol. Sci.* **2020**, *173* (1), 202–225.
(15) Mansouri, K.; Karmaus, A. L.; Fitzpatrick, J.; Patlewicz, G.; Pradeep, P.; Alberga, D.; Alepee, N.; Allen, T. E. H.; Allen, D.; Alves, V. M.; Andrade, C. H.; Auernhammer, T. R.; Ballabio, D.; Bell, S.; Benfenati, E.; Bhattacharya, S.; Bastos, J. V.; Boyd, S.; Brown, J. B.; Capuzzi, S. J.; Chushak, Y.; Ciallella, H.; Clark, A. M.; Consonni, V.; Daga, P. R.; Ekins, S.; Farag, S.; Fedorov, M.; Fourches, D.; Gadaleta, D.; Gao, F.; Gearhart, J. M.; Goh, G.; Goodman, J. M.; Grisoni, F.; Grulke, C. M.; Hartung, T.; Hirn, M.; Karpov, P.; Korotcov, A.; Lavado, G. J.; Lawless, M.; Li, X.; Luechtefeld, T.; Lunghini, F.; Mangiatordi, G. F.; Marcou, G.; Marsh, D.; Martin, T.; Mauri, A.; Muratov, E. N.; Myatt, G. J.; Nguyen, D.-T.; Nicolotti, O.; Note, R.; Pande, P.; Parks, A. K.; Peryea, T.; Polash, A. H.; Rallo, R.; Roncaglioni, A.; Rowlands, C.; Ruiz, P.; Russo, D. P.; Sayed, A.; Sayre, R.; Sheils, T.; Siegel, C.; Silva, A. C.; Simeonov, A.; Sosnin, S.; Southall, N.; Strickland, J.; Tang, Y.; Teppen, B.; Tetko, I. V.; Thomas, D.; Tkachenko, V.; Todeschini, R.; Toma, C.; Tripodi, I.; Trisciuzzi, D.; Tropsha, A.; Varnek, A.; Vukovic, K.; Wang, Z.; Wang, L.; Waters, K. M.; Wedlake, A. J.; Wijeyesakere, S. J.; Wilson, D.; Xiao, Z.; Yang, H.; Zahoranszky-Kohalmi, G.; Zakharov, A. V.; Zhang, F. F.; Zhang, Z.; Zhao, T.; Zhu, H.; Zorn, K. M.; Casey, W.; Kleinstreuer, N. C. CATMoS: Collaborative Acute Toxicity Modeling Suite. *Environ. Health Perspect.* **2021**, *129* (4), 047013. https://doi.org/10.1289/EHP8495.
(16) Pedregosa, F.; Varoquaux, G.; Gramfort, A.; Michel, V.; Thirion, B.; Grisel, O.; Blondel, M.; Prettenhofer, P.; Weiss, R.; Dubourg, V. Scikit-Learn: Machine Learning in Python. *J. Mach. Learn. Res.* **2011**, *12*, 2825–2830.
(17) Organisation for Economic Co-operation and Development (OECD). *(Q)SAR Assessment Framework: Guidance for the Regulatory Assessment of (Quantitative) Structure - Activity Relationship Models, Predictions, and Results Based on Multiple Predictions, OECD Series on Testing and Assessment, No. 386, Environment, Health and Safety, Environment Directorate, OECD*.
